# Intracranial aneurysm wall (in)stability–current state of knowledge and clinical perspectives

**DOI:** 10.1007/s10143-021-01672-5

**Published:** 2021-11-06

**Authors:** Sandrine Morel, Philippe Bijlenga, Brenda R. Kwak

**Affiliations:** 1 Department of Pathology and Immunology, Faculty of Medicine, Centre Medical Universitaire, University of Geneva, Rue Michel-Servet 1, 1211 Geneva, Switzerland; 2 Neurosurgery Division, Department of Clinical Neurosciences, Faculty of Medicine, Geneva University Hospitals and University of Geneva, Geneva, Switzerland

**Keywords:** Intracranial aneurysm, Subarachnoid hemorrhage, Risk factors, Vessel wall, Hemodynamics, Magnetic resonance imaging

## Abstract

Intracranial aneurysm (IA), a local outpouching of cerebral arteries, is present in 3 to 5% of the population. Once formed, an IA can remain stable, grow, or rupture. Determining the evolution of IAs is almost impossible. Rupture of an IA leads to subarachnoid hemorrhage and affects mostly young people with heavy consequences in terms of death, disabilities, and socioeconomic burden. Even if the large majority of IAs will never rupture, it is critical to determine which IA might be at risk of rupture. IA (in)stability is dependent on the composition of its wall and on its ability to repair. The biology of the IA wall is complex and not completely understood. Nowadays, the risk of rupture of an IA is estimated in clinics by using scores based on the characteristics of the IA itself and on the anamnesis of the patient. Classification and prediction using these scores are not satisfying and decisions whether a patient should be observed or treated need to be better informed by more reliable biomarkers. In the present review, the effects of known risk factors for rupture, as well as the effects of biomechanical forces on the IA wall composition, will be summarized. Moreover, recent advances in high-resolution vessel wall magnetic resonance imaging, which are promising tools to discriminate between stable and unstable IAs, will be described. Common data elements recently defined to improve IA disease knowledge and disease management will be presented. Finally, recent findings in genetics will be introduced and future directions in the field of IA will be exposed.

## Introduction

Three to 5% of the population hold an unruptured intracranial aneurysm (IA) [98]. IAs, which result from the deformation and the enlargement of the arterial lumen, have usually a saccular form and are most often observed at bifurcations of cerebral arteries in the circle of Willis [15, 57]. The natural evolution of an unruptured IA may be its rupture immediately after its formation, its growth before rupturing, or it can remain stable. Predicting the evolution of an unruptured IA is very complex. The annual rupture rate of an IA is around 1%, inducing a prevalence of aneurysmal subarachnoid hemorrhage (SAH) of 9 to 100.000 inhabitants per year [49]. SAH is a devastating form of stroke associated with a high level of mortality (50%) and morbidity causing dependence (30–50%) [80]. As SAH affects mainly individuals 40 to 60 years old, its socioeconomic burden is heavy. Due to the increased use of high-quality radiologic imaging, an increasing number of unruptured IAs are diagnosed, confronting clinicians and patients more and more often to the difficult question whether to treat or not an unruptured IA. Procedural morbidity and mortality to occlude an unruptured IA by microsurgical or endovascular treatment is around 2 to 5% [19]. Every time an unruptured IA is discovered, the risk of mortality and morbidity associated with the rupture of the IA has to be balanced with the incurred risks associated with its treatment.

In 2014 and 2015, two scores evaluating the risk of rupture of an IA have been published to help clinicians in their decision to observe or to treat an unruptured IAs: (1) the PHASES score [28] predicting the 5-years risk of IA rupture based on 6 sets of data routinely assessed in clinic: population, hypertension, age, size of the aneurysm, early SAH from another IA, and the site of IA (Table 1), and (2) the unruptured IA treatment (UIAT) score [18] accounting for 29 key factors on patients, IAs, and considering the risk associated with treatment (Table 1). In 2017, Backes et al. [1] proposed the ELAPSS score to predict the risk of growth based on 6 predictors (Table 1). Since their creation, the validity of these 3 scores has been tested in different cohorts showing that the PHASES, UIAT, or ELAPSS scores are indeed valuable for clinicians to help them in their decision to observe or treat an unruptured IAs, but they also highlight that additional information is needed to sustain decision [4, 6, 7, 20, 35, 64, 72, 77, 86]. In the first part of this review, the effects of some risk factors used in these three scores on the aneurysm wall composition will be presented. Aneurysm wall (in)stability is also influenced by biomechanical forces; their effects will be described in the second part of the review. As demonstrated in the different validation studies, the specificities and sensitivities of the three scores are not strong. Additional clinical factors that might be considered in the decision to intervene or not, such as radiological vessel wall imaging, additional clinical information, or patient’s genetic profile, will be described in the third, fourth, and fifth parts of this review. Finally, tools under development to better define IA (in)stability and help clinical decision-making will be briefly presented in the last part of this review.

**Table 1:** Clinical scores determining IA risk of rupture or growth (adapted from Greving et al. [28], Etminan et al. [18], and Backes et al. [1])

	PHASES score [28]	UIAT score [18]	ELAPSS score [1]
Aneurysm	- Size of the aneurysm (< 7.0 mm, 7.0–9.9 mm, 10.0–19.9 mm, ≥ 20.0 mm)	- Maximum diameter (≤ 3.9 mm, 4.0–6.9 mm, 7.0–12.9 mm, 13.0–24.9 mm, ≥ 25.0 mm)	- Size of the aneurysm (1.0–2.9 mm, 3.0–4.9 mm, 5.0–6.9 mm, 7.0–9.9 mm, ≥ 10.0 mm)
	- Site of IA (ICA, MCA, ACA/Pcom/posterior)	- Location (BasA bifurcation, vertebral/basilar artery, AcomA or PcomA)	- Location of the IA (ICA/ACA/Acom, MCA, Pcom/posterior)
		- Morphology (irregularity or lobulation, size ratio > 3 or aspect ratio > 1.6) - IA growth on serial imaging - IA de novo formation on serial imaging - Contralateral steno-occlusive vessel disease	- Shape of the IA (regular or irregular)
Patient	- Age (< or ≥ 70 years)	- Age (< 40 years, 40–60 years, 61–70 years, 71–80 years, > 80 years)	- Age (≤ or > 60 years)
	- Early SAH from another IA (yes/no) - Population (North American, European (other than Finnish), Japanese, Finnish) - Hypertension (yes/no)	- Risk factor incidence (previous SAH from a different IA, familial IA or SAH, Japanese/Finnish/Inuit ethnicity, current cigarette smoking, hypertension, APKD, current drug and/or alcohol abuse)	- Earlier SAH (yes/no) - Population (North American, China, Europe (other than Finland), Japan, Finland)
		- Clinical symptoms related to unruptured IA (cranial nerve deficit, clinical or radiological mass effect, thromboembolic events from the IA, epilepsy) - Reduced quality of life due to fear of rupture - Aneurysm multiplicity - Life expectancy due to chronic and/or malignant diseases (< 5 years, 5–10 years, < 10 years) - Comorbid diseases (neurocognitive disorder, coagulopathies, thrombophilic diseases, psychiatric disorder)	
Treatment		- Age-related risk (< 40 years, 40–60 years, 61–70 years, 71–80 years, > 80 years) - Aneurysm size-related risk (< 6.0 mm, 6.0–10.0 mm, 10.1–20.0 mm, > 20.0 mm) - Aneurysm complexity-related risk (high/low) - Intervention-related risk (constant)	

*ACA* anterior cerebral artery, *AcomA* anterior communicating artery, *APKD* autosomal-polycystic kidney disease, *BasA* basilar artery, *IA* intracranial aneurysm, *ICA* internal carotid artery, *MCA* middle cerebral artery, *Pcom* posterior communicating artery, *SAH* subarachnoid hemorrhage

## How do risk factors for intracranial aneurysm rupture or growth affect aneurysmal wall organization?

Cerebral arteries have a specific organization with loss of external elastic lamina and thinner supporting adventitial tissue [94]. IA formation is initiated by the disruption of the internal elastic lamina followed by the remodeling of the vessel wall. Processes of IA wall remodeling and degeneration have been extensively described in recent reviews [93, 94]. Briefly, during wall remodeling, extracellular matrix (ECM) turnover is balanced between its production by smooth muscle cells (SMCs) undergoing a phenotypic modulation from contractile to synthetic type, and its degradation by metalloproteinases (MMPs) secreted by inflammatory cells. Thus, aneurysmal wall degeneration and weakness leading to its rupture is linked to a high inflammatory cell infiltration, disappearance of SMCs, lipid accumulation, and calcification. Several classifications of the aneurysmal wall from the little to high risk of rupture have been proposed. In 1999, Kataoka et al. [41] have proposed three scores characterizing the integrity of the inner surface of the aneurysmal sac (Table 2), the structure of the aneurysmal wall (Table 3), and the inflammatory cell invasion into the aneurysmal wall (Table 4). In 2004, Frösen et al. [21] proposed a unique classification taking into account the entire aneurysmal wall (Table 5). In our cohort of patients treated in the Geneva University Hospitals (the Swiss AneuX study) [55], we sorted 48 IAs according to this classification, and we showed that ruptured IAs present a higher percentage of wall type C and D, i.e., degenerative histological phenotypes, in comparison to unruptured IAs. More recently, Gade et al. [24] have proposed a new classification according to calcification and lipid pools (Table 6).

**Table 2:** Aneurysmal sac inner surface score (adapted from Kataoka et al. [41])

Score	Histological observations
0	Normal endothelial cell layer inside the aneurysmal sac, possible adhesion of few leukocytes
1	Endothelial cells with various shapes, intercellular filaments and wider intercellular gaps
2	Damaged endothelial cell layer at some locations with blood cells adhesion
3	Extended damages of the endothelial cell layer and more blood cells adhesion
4	Extensive endothelial cell layer damages with blood cells adhesion
5	Almost entire endothelial cell layer damaged and covered with blood cells and a fibrin network

**Table 3:** Aneurysmal structural wall score (adapted from Kataoka et al. [41])

Score	Histological observations
1	Dense wall with smooth muscle cells and regular layers of type IV collagen
2	Dense wall with smooth muscle cells and irregular layers of type IV collagen or scattered smooth muscle cells and relatively regular layers of type IV collagen
3	Scattered smooth muscle cells and irregular layers of type IV collagen
4	Presence of hyaline-like structures. Smooth muscle cells and collagen are still present
5	Hyaline-like structures in the entire aneurysmal wall. Smooth muscle cells and collagen are absent

**Table 4:** Aneurysmal wall inflammatory cell invasion score (adapted from Kataoka et al. [41])

Score	Histological observations
0	Presence of few macrophages. Absence of positive staining for cathepsin G. Cathepsin D signal can be observed
1	Presence of macrophages. Cathepsin D is detected
2	Presence of macrophage clusters. Strong signal for cathepsin D. Scattered smooths muscle cells and disrupted collagen layer. Presence of leukocytes
3	Diffuse invasion of macrophages in the aneurysmal wall. Strong signal for cathepsin D may be present. Presence of leukocytes and eventual signal for cathepsin G

**Table 5:** Aneurysm wall type classification (adapted from Frösen et al. [21])

Wall type	Histological observations
A	Endothelialized wall and linearly organized smooth muscle cells
B	Thickened wall with disorganized smooth muscle cells
C	Hypocellular wall with either intimal hyperplasia or organized luminal thrombosis
D	Extremely thin thrombosis-lined hypocellular wall

**Table 6:** Wall classification according to the presence of calcification and lipid pools (adapted from Gade et al. [24])

Wall type	Histological observations
I	Wall containing calcification without any lipid pools
II	Wall containing both calcification and lipid pools though never co-localized
III	Wall containing calcification co-localized with lipid pools

These different classifications clearly show that IA wall integrity is dependent on the presence and on the properties of endothelial cells (ECs), SMCs and inflammatory cells, and on the homogeneity/heterogeneity of the IA wall. Presence and properties of these cells can be affected by the different risk factors for IA rupture and growth used in the clinical scores described above. The effects of some of these risk factors on the IA wall composition have been investigated in human IAs or in experimentally induced IAs in animals by performing histological analyses. The results of such studies are described below.

### Aneurysm morphology characteristics and intracranial aneurysm wall composition

Irregularities in IA wall morphology have been associated with wall instability [22, 38, 55]. Links between IA morphology characteristics and activation of intracellular pathways have been investigated in different studies. For example, presence of phospho-mitogen-activated protein kinases (MAPKs) has been demonstrated in SMCs of ruptured and unruptured IAs [45]. MAPKs are a family of intracellular signaling proteins playing an important role in cell growth, proliferation, differentiation, and death. MAPK family is constituted of c-jun N-terminal kinases (JNKs), p38 MAPKs, and extracellular signal-regulated kinases (ERKs). To be activated, MAPKs have to be phosphorylated by upstream kinases. Laaksamo et al. [45] have shown that ruptured IAs have a higher content of phosphorylated p54 JNK whereas other MAPKs are not affected. Currently, IA maximal diameter is the main anatomic factor used by clinicians to assess the risk of rupture. Interestingly, covariance analysis showed that the level of phospho-p54 JNK, or the level of total p38, was associated with IA size but not with rupture status. The level of phospho-p38 was associated with IA size and rupture status. No correlation between IA size and ERK levels has been found. Moreover, sex or age of the patients does not affect the level of MAPKs phosphorylation [45]. In addition to maximal IA size, other size indexes can be used such as fundus length, neck diameter, IA volume, or surface area. Moreover, shape indexes such as aspect ratio (fundus length/neck diameter), bottleneck factor (maximum dome diameter/neck diameter), bulge location (distance from neck to maximum dome diameter as a fraction of fundus length), undulation index (presence of irregularities, lobulations, or daughter sacs), ellipticity index (deviation of the IA shape from that of a perfect hemisphere), and non-sphericity index (aggregate of undulation index and ellipticity index) can also help to determine the risk of rupture of an IA. Laaksamo et al. [44] found associations between some of these indexes and total and/or phosphorylated levels of JNK or p38. Moreover, they analyzed downstream targets of MAPKs. Their main observations were that maximal IA size was positively correlated with levels of phospho-p54 JNK, phospho-p38, and phospho-Akt, that ellipticity index and non-sphericity index were associated with phospho-CREB (c-AMP response element-binding protein) levels, and that undulation index was negatively correlated with phospho-p38 and phospho-Akt. As CREB is a regulator of cell growth and proliferation, its activation may contribute to intimal hyperplasia. Akt is a cell survivor promoter which can be involved in protective repair processes. No associations between morphological indexes and the apoptosis regulator Bad or the cell-cycle regulator mTOR have been found [44]. Altogether, these results suggest that p54 JNK and p38 and their downstream activation pathways are involved in IA remodeling. More investigations are needed to determine whether this activation results in the weakening or in the repair of the IA wall. With respect to cell death, no association has been found between size indexes and cleaved caspase-9. Expression of heme oxygenase-1, which is a detoxification enzyme induced by oxidative stress, is correlated with fundus length and aspect ratio, independently of the rupture status [46]. These results suggest a link between IA morphology and oxidative stress level. In our study performed on 31 unruptured IA human domes resected after clipping, we have shown a positive correlation between IA maximum dome diameter and inflammatory cell infiltration and collagen content [55]. Concerning the radiological surface aspect of the IA, we found that the wall of rough IAs has a higher content in type III collagen in comparison to IAs with a smooth radiological aspect, leading to a change in the ratio type I/type III collagen [55]. Type I collagen fibers have been shown to be located in the adventitial layer of cerebral arteries whereas type III collagen fiber are mostly found in the media [74]. In the aneurysmal wall, different collagen fiber diameters have also been shown between the luminal and the abluminal parts of the wall, and the collagen layer at the luminal side was different between IAs and control cerebral arteries suggesting difference in the collagen fibers turnover [74]. However, comparison of collagen organization and IA wall strength showed a large variability between different types of unruptured IAs. Therefore, the consequences of collagen organization on IA morphology need further investigation.

### Ethnicity and intracranial aneurysm wall composition

Although Japanese or Finnish ethnicity seems to be an important risk factor for aneurysmal disease, no systematic study comparing the effects of different ethnicities for aneurysmal wall composition has been done so far. Comparison of the various studies performed in cohorts from different ethnicities is difficult due to absence of a research consortium for the histological characterization of the IA wall. In Table 7, histological characterization of aneurysmal wall from Japanese [41], Finnish [21], and Swiss [55] population is presented. Although there may be slight differences between the three cohorts, the overall comparison between these three studies on different ethnicities confirms once more that ruptured IAs present a more degenerative wall in comparison to the wall of unruptured IAs. The degeneration process affects first the endothelium, then the structure of the vascular wall and finally favors the progression of inflammation. However, even if the mechanisms of IA wall degeneration seem nearby between the three cohorts, cases selection, wall classification, and methods of histological quantifications are different between the cohorts which could reduce the chances to observe differences between ethnicities.

**Table 7:** Histological characteristics of aneurysmal wall from 3 cohorts of patients coming from Japan, Finland and Switzerland. The values come from the articles published by Kataoka et al. [41] (Japanese patients), Frösen et al. [21] (Finnish patients), and Morel et al. [55] (Swiss patients). Definitions of each score or wall type are given in Tables 2, 3, 4, 5

Cohorts and wall characteristics	Unruptured IA	Ruptured IA
Japanese patients	*N* = 27	*N* = 44
Aneurysmal sac inner surface score	0.8	3.7
Aneurysmal structural wall score	1.7	3.7
Aneurysmal wall inflammatory cell invasion score	0.8	2.2
Finnish patients	*N* = 24	*N* = 42
Endothelial lining absent	7/23 (30%)	25/40 (62%)
Wall type A	10 (42%)	7 (17%)
Wall type B	9 (37%)	9 (21%)
Wall type C	5 (21%)	11 (26%)
Wall type D	0 (0%)	15 (36%)
Swiss patients	*N* = 31	*N* = 17
Endothelial cells covering the aneurysmal wall	2.2 to 91.1%	7.1 to 52.8%
Wall type A	5 (16%)	0 (0%)
Wall type B	17 (55%)	3 (18%)
Wall type C	8 (26%)	12 (70%)
Wall type D	1 (3%)	2 (12%)

### Smoking and intracranial aneurysm wall composition

Smoking, a risk factor for IA formation and rupture, is known to increase oxidative stress. Oxidative stress participates to several pathological pathways involved in vessel wall degeneration such as endothelial dysfunction, inflammatory cell infiltration, SMC phenotypic change, and cell death (for a review see [10]).

The existence of endothelial dysfunction has been clearly demonstrated in many vascular pathologies including IA. Cigarette smoke has been described to favor endothelial dysfunction in cerebral arteries [10]. By activating caspase-3, cigarette smoke favors endothelial cell death. Moreover, cigarette smoke increases the pro-inflammatory properties of ECs, promoting adherence and migration of inflammatory cells inside the aneurysmal wall. In particular, nicotine favors inflammatory cell infiltration by decreasing the expression of tight junction proteins such as zonula occludens-1, occludin, cadherin, or adherence junction proteins [10]. Surprisingly, Ollikainen et al. [62] have shown that the aneurysmal wall of smokers contains less macrophages (CD68- and CD163-positive cells) and less apolipoprotein A-I than the wall of non-smokers. In their study, smoking was also not associated with lipids, oxidized lipids, or apolipoprotein B-100 accumulation in the aneurysmal wall [62].

**Fig. 1:**
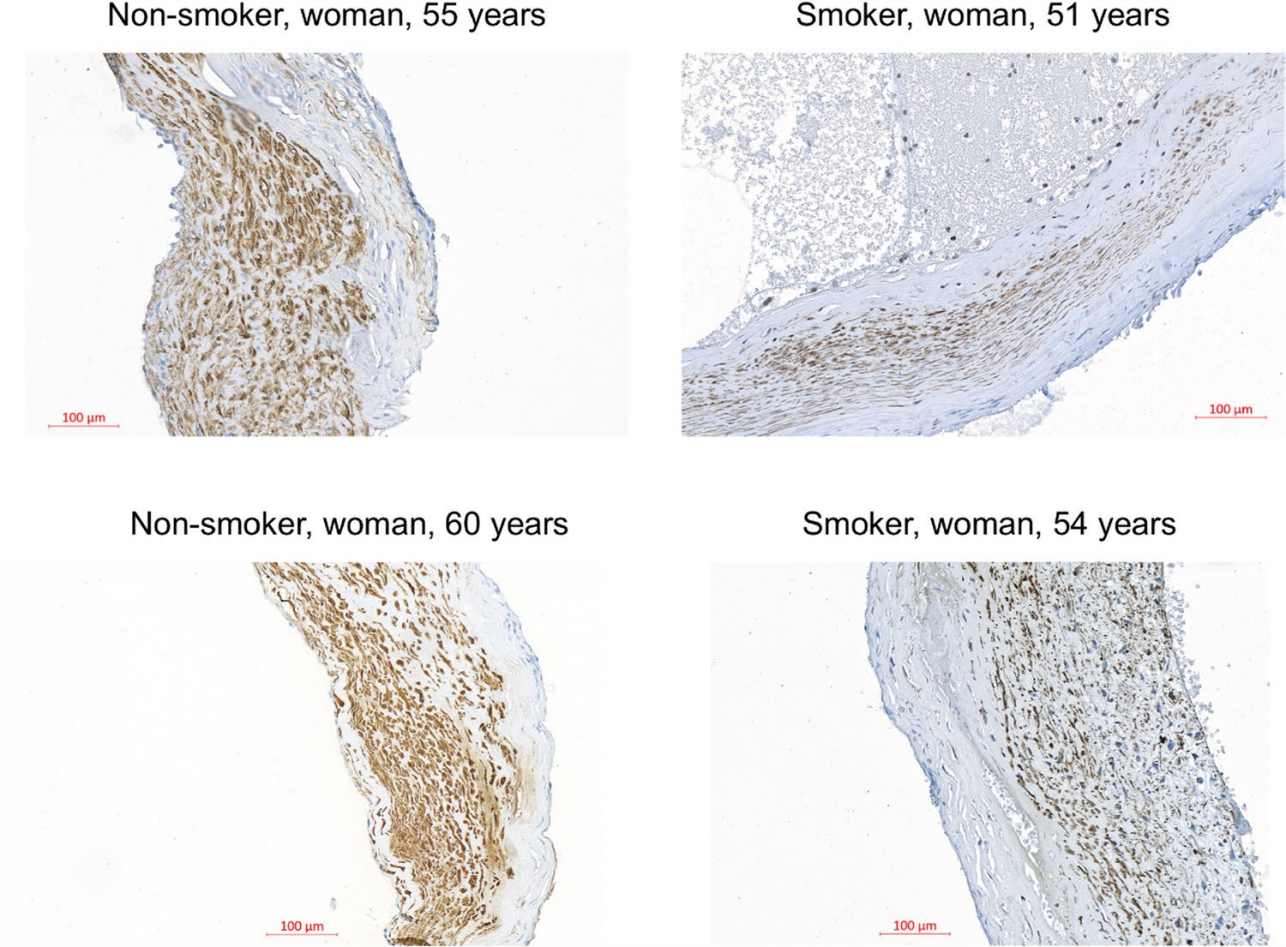
Aneurysmal wall of smokers has a lower content in smooth muscle cells in comparison to the wall of non-smokers. Representative images of the wall of four IAs stained with alpha-smooth muscle actin to visualize smooth muscle cells in brown. Human saccular IA samples were obtained during microsurgery by resecting the aneurysmal dome after clipping of the aneurysmal neck. The four IAs coming from the Swiss AneuX biobank were located on the middle cerebral artery of two non-smoker (left images) and two smoker (right images) patients. Scale bar = 100 um

Phenotypic switch and apoptosis of SMCs are favored by oxidative stress and reactive oxygen species (ROS). Using cultured cerebral SMCs, Starke et al. [89] has shown that cigarette smoke exposure increased nicotinamide adenine dinucleotide phosphate oxidase (NOX) 1 expression, favored ROS production, upregulated pro-inflammatory and matrix remodeling genes, and downregulated SMC contractile genes. In a mouse model of IA induced by elastase treatment, they showed that exposure of the mice to cigarette smoke increased the number of IA ruptures, and that the inhibition of NOX1 reduced this incidence of rupture [89]. Finally, the authors showed that IAs from mice in which NOX1 expression or activity was reduced harbored a higher content of SMCs contractile markers. Interestingly, such SMC contractile markers were also reduced in normal arteries of mice exposed to cigarette smoke, while inflammatory and matrix remodeling genes were upregulated [89]. In a cohort of 31 patients with unruptured IAs, we showed that smokers present a lower content of SMCs in their aneurysmal wall compared to non-smokers [55] (Fig. 1). Remarkably, this reduced SMC content is close to the one measured in ruptured IAs. Smoking has been demonstrated to increase MMPs and to reduce collagen synthesis [10], which may result in aneurysmal wall thinning and rupture. Heme oxygenase-1 expression is reduced in smokers [46], which suggests that smoking affects the protective mechanisms against oxidative stress thereby favoring cell death. Moreover, SMC injury and death has also been proposed to be due to the upregulation of calcium channels [26]. Altogether, these studies suggest that smoking favors SMC death and aneurysmal wall degeneration.

### Hypertension and intracranial aneurysm wall composition

Hypertension has been associated with physiological and morphological changes in the vasculature. Similar to smoking, hypertension seems to increase oxidative stress. However, the direct link between hypertension, ROS, and IA development is not obvious, as heme oxygenase-1 expression is downregulated in patients with hypertension [46]. Using mathematical modeling on the basis of ^14^C birth dating, Hackenberg et al. [31] have evaluated in a recent study the collagen turnover rate and the collagen mean age in the wall of IAs. They showed that the mean collagen turnover rate was higher than 2600% per year and the mean collagen age was less than 2 weeks in hypertensive patients, whereas in patients without hypertension the turnover rate was 55% per year with a mean age of collagen 1.8 years. On histological sections, they observed rather unstructured and immature collagen fibers in patients with arterial hypertension and rather structured and mature collagen fibers in patients without this risk factor. More investigations are needed to determine by which mechanisms hypertension affects collagen degradation and synthesis.

### Polycystic kidney disease and intracranial aneurysm wall composition

People affected by polycystic kidney disease (PKD) are more prone to develop IAs than the general population with a prevalence of IAs ranging from 4 to 40% [8]. In addition, individual IAs of PKD patients seem to be more prone to rupture. PKD is characterized by an absence or lack of function in primary cilia. In our Swiss AneuX cohort of IA domes harvested after micro-surgery, we have performed histological analysis to compare the aneurysmal wall composition of unruptured IA domes from PKD patients with unruptured IA domes from non-PKD patients. We showed that IA walls from PKD patients were thinner compared to the IA wall from non-PKD patients and that their collagen content was lower [16]. Following the classification described in Table 5, we showed that IA domes from PKD patients showed a higher frequency of the most severe phenotype (grade D) than non-PKD IA domes [16]. By comparing primary cilia-deficient ECs with ECs expressing normal primary cilia, we demonstrated that absence of primary cilia is associated with reduced barrier integrity concomitant with disruption of proteins comprising different intercellular junctions, such as zonula occludens-1 and -2, catenin α-1 and β-1, connexin43 and claudin-3 [16].

### Sex and intracranial aneurysm wall composition

The relationship between sex and IA risk of rupture is not clear and controversial. Although the different scores described above do not include sex as a risk factor for IA rupture, women are known to be more at risk for IA formation and growth than men [27]. Hormonal and hemodynamic changes are both believed to be responsible of such observations. As women at post-menopausal age have a higher prevalence of IAs, the importance of sex hormone in IA formation, growth, and rupture has been suggested. By using a surgical model of grafted side-wall aneurysms in female, male, and ovariectomized rats, we showed sex-related differences in IA wall remodeling and intraluminal thrombus resolution [56]. In particular, we showed in female and ovariectomized rats that aneurysmal wall inflammation was lower in regular aneurysms in comparison to aneurysms that have been decellularized before grafting, whereas in male, wall inflammation was larger in regular aneurysms than in decellularized ones. Although estrogens are usually considered to be anti-inflammatory, androgens can have anti- or pro-inflammatory properties depending on the pathophysiological status of the patient (e.g., existence of a cerebral disease, age, hormonal levels). The differences in inflammatory cell infiltration in the aneurysmal wall may also be attributed to different wall shear stress values between females and males. General thrombus organization was similar between female, male, and ovariectomized rats with the exception of collagen content. Indeed, collagen content was higher in male in comparison to female rats [56], a phenomenon that was also observed in patients suffering from abdominal aortic aneurysms. Estrogen is known to reduce collagen deposition in human arteries. Interestingly, aneurysm growth was larger in ovariectomized rats in comparison to female or male rats. In a model of ovariectomized mice, Tada et al. [91] have reported a protective effect of estrogen against aneurysmal growth and rupture. Moreover, androgen excess in women has been proposed to be associated with endothelial dysfunction [96]. In our model, we showed that the intraluminal EC coverage of the thrombus was lower in ovariectomized rats in comparison to female or male rats, which may explain the observed differences in aneurysm growth. Analysis of our human IAs cohort showed that IA domes from men or post-menopausal women were less covered by ECs than IA domes of pre-menopausal women [56]. Estrogen contributes to vascular integrity by regulating inflammatory cascades. Lack of estrogen may favor aneurysmal wall degeneration and explain the higher IA growth in post-menopausal women. In a study involving 31 women and 16 men with a mean age of 54 years, Kadasi et al. [40] have shown by semiquantitative wall thickness measurement performed on intraoperative images that the proportion of super-thin translucent tissue in IAs, i.e., IA wall regions at risk of rupture, was higher in women in comparison to men. In rats, Wang et al. [101] showed that the vascular wall of middle cerebral arteries was thinner in females in comparison to males. They also showed that the arterial wall of females had less SMCs and more collagen content than males. Such type of differences may contribute to the higher prevalence of IA formation and growth in women. With respect to IA rupture, a recent study performed by Oka et al. [59] showed that IA rupture rate in ovariectomized female rats was higher than in female or male rats, confirming the crucial role of estrogen in IA rupture. Interestingly, the authors demonstrated that inflammatory cell infiltration was similar between unruptured IAs in ovariectomized female rats and all observed ruptured IAs, suggesting an exacerbation of the disease in these animals. Using an additional model of carotid stenosis, they further demonstrated that EC function was disturbed in female rats with bilateral ovariectomy indicating a protective role of estrogen in EC function. In combining these two models in their study, the authors have linked the effect of female sex on IA disease progression and rupture with inflammation and EC dysfunction.

### Effects of known risk factors for vascular diseases not included in the PHASES, UIAT, or ELAPSS scores

Lipids are important contributors to vascular diseases. Despite normal plasma cholesterol and triglyceride levels, lipid accumulation may be observed in IA walls, an event that is associated with IA wall degeneration [94]. In a rat model of induced IA, Shimuzu et al. [84] have shown that high-fat diet favored the loss of SMCs in the aneurysmal wall and the enlargement of the IAs. They found lipid accumulation in half of the IAs of rats on a high-fat diet. However, these degenerative changes were not associated with a higher rate of IA rupture. Presence of oxidized lipids in the vessel wall is known to induce formation of acquired antibodies, which in turn seems to be involved in the clearing of oxidized lipids from the vessel wall. Patients with unruptured IA have been shown to have a higher plasma levels of oxidized lipids reactive IgGs than patients with SAH [94]. The authors suggested that acquired immunity against oxidized lipids may be protective against the IA wall degeneration associated with lipid accumulation.

Oral and gut microbiota emerged as critical environmental factors contributing to human physiology and pathology. Recently, oral bacteria–derived DNA has been found in both ruptured and unruptured IA walls in surgically treated Finnish patients [67, 68], suggesting that dental infection could be a part of the pathophysiology of IA disease. Moreover, a mouse model of induced IA formation showed the relevance of gut microbiota for IA formation and showed that exposure to gut microbiota affects the remodeling of cerebral artery wall through modulation of local immune response without local bacterial infection [81]. While the mechanisms how periodontitis predisposes to IA formation still remains unclear, potential mechanisms include (i) activation of Toll-like receptors in the artery wall by bacteria-derived components in the bloodstream, (ii) activation of circulating neutrophils by bacteria-derived components in the bloodstream, and (iii) development of an immunological memory that reacts to bacteria-derived particles, or through molecular mimicry to other epitopes in the cerebral artery wall [81]. Interestingly, Hallikainen et al. [32] have shown that IA patients use more antibiotics than their age- and gender-matched population, suggesting that dysbiosis of the microbiota might be relevant in IA formation. While it remains to be studied whether gastrointestinal tract microbiota is relevant for IA formation in humans, it is important to note that oral microbiota dysbiosis can affect also the microbiota of the gastrointestinal tract [63], thus generating yet another possible explanation how periodontitis may predispose to IA formation. Oral and intestinal microbiota are strongly influenced by the local environment and eating habits and would represent a new factor influencing IA disease. Importantly, identification of specific forms of oral and gut microbiota would serve as risk biomarkers for IA disease and rupture, and as a target for medical treatment.

### Aneurysmal wall components not affected by risk factors for intracranial aneurysm rupture or growth

Mast cells have been found in the aneurysmal wall but the presence of these regulators of both innate and adaptive immune systems was not associated with age, sex, smoking, hypertension, previous SAH, IA size, or IA multiplicity [60]. Myeloperoxidase, a marker for neutrophil infiltration, which is associated with IA degenerative remodeling and IA rupture, seems also not correlated with clinical risk factors such as IA size, fundus length, fundus width, or smoking [61]. However, divergent results have been obtained for the correlation between myeloperoxidase content in IA wall and aneurysm rupture risk calculated with the PHASES score. Finally, the PHASES score was not associated with lipids, oxidized lipids, or adipophilin accumulation in the aneurysmal wall, but was inversely correlated with apolipoprotein A-I content at this location [62].

### Limitations of histological studies

Human and animal histological investigations have brought a lot of information about IA wall evolution throughout IA life and how risk factors influence this evolution. Nevertheless, it should not be forgotten that histological investigations done on human aneurysm domes are performed only on IA harvested after microsurgery, representing only a part of discovered IAs. An important bias in such histological investigations is, although IA location is a very important factor balancing the individual risk of rupture [18, 28, 75], no study has investigated IA wall composition depending on the arterial location. Moreover, only a limited part of the IAs is available for the histological studies observations, and this part may not reflect the characteristics of entire IA wall. Thus, one should be cautious and critical when interpreting the associations between clinical risk factors for growth and rupture and histopathological findings.

### Perspectives

To better determine the effects of risk factors on IA wall composition, more studies are needed and a consortium with standard protocols for sample processing and histological analyses is required. In 2019, the unruptured IAs and SAH Common Data Elements (CDEs) project has led to the publication of 8 articles defining CDEs to standardize clinical and fundamental research in the field of IAs (see below in the paragraph: Common data elements to improve the current state of knowledge). More specifically, Chou et al. [11] have proposed standard operating procedure guideline for biospecimen harvesting which would uniformize data collection and improve data quality collection throughout the different hospitals or research groups, and would allow for better comparison between studies. Importantly, national initiatives for research infrastructures supporting the quality assessment and normalization of human and non-human biobanks are currently developed, including in Switzerland (https://swissbiobanking.ch/).

## How do biomechanical forces influence intracranial aneurysm wall (in)stability?

**Fig. 2:**
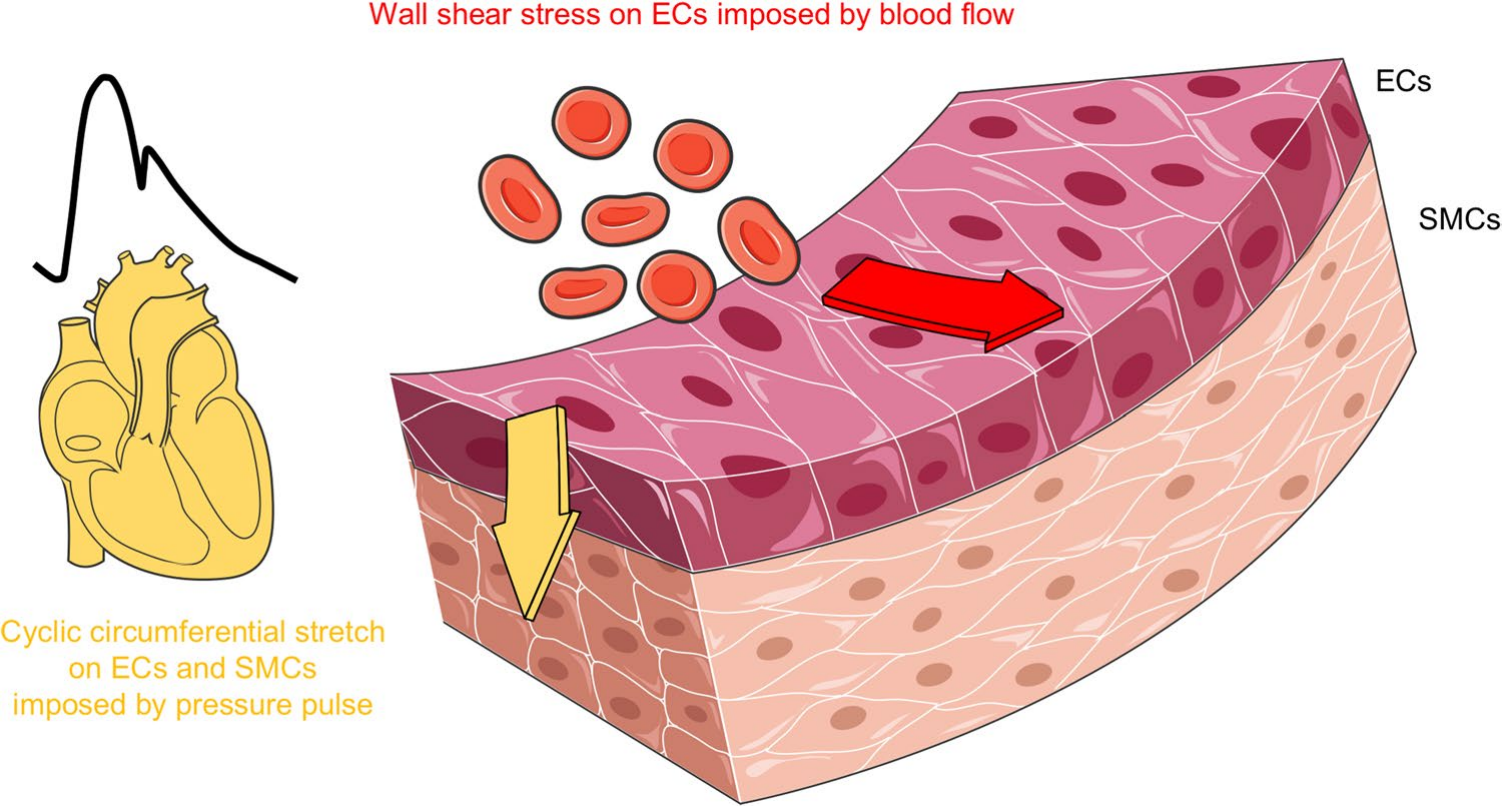
Biomechanical forces acting on the arterial wall. Wall shear stress imposed by the flowing blood on the wall is defined as the tangential force per unit area (red arrow). Wall shear stress is sensed by endothelial cells (ECs). Cyclic circumferential stretch is the perpendicular force imposed by the pressure pulse on the vessel wall (yellow arrow). Cyclic circumferential stretch is sensed by ECs and smooth muscle cells (SMCs)

Hemodynamical forces are considered to be important players of IA formation, remodeling, and rupture. Two types of biomechanical forces act on the arterial wall: wall shear stress (WSS) defined as the tangential force imposed by the flowing blood on the wall per unit area, and a cyclic strain caused by the intravascular pressure called cyclic circumferential stretch (CCS). In the arterial wall, sensors of WSS are ECs and sensors of CCS are both ECs and SMCs (Fig. 2).

### Biomechanical forces in intracranial aneurysm

Physiological stretch participates to vascular maintenance by activating pathways involved in proliferation, angiogenesis, ROS formation, vascular tone, and vascular remodeling [39]. Excessive stretch, as observed during hypertension, leads to inappropriate cellular responses such as increased inflammation, exacerbated ROS production, EC apoptosis, alteration of migratory and proliferative cellular activity, or ECM reorganization. In the context of IA, Liu et al. [50] showed that exposure to 15% CCS reduced SMC viability and invasive capacity. Moreover, the expression of collagen types IV and VI were downregulated and MMP expression was upregulated. In human, they also showed a sparser SMC distribution in IAs in comparison to normal superficial arteries, and a lower expression of the two collagen types. In a model of elastase-induced rabbit aneurysms, increased longitudinal load has been shown to trigger a vascular remodeling response following two distinct phases [78]. The first phase occurring quickly after aneurysm induction (2 weeks) was characterized by the appearance of sparse and multi-directional collagen fibers at the interface between the media and the adventitia. The authors proposed that such development provides a mechanism for the aneurysm wall to bear longitudinal and circumferential loads without extensive elongation. Few months after aneurysm induction, a reorganization of the medial collagen fibers was observed. Such remodeling, which is comparable to the one reported in humans [74, 78], provides a possible mechanism by which IAs can adapt to changes in mechanical load.

Physiological flow present in the straight part of an artery corresponds to a unidirectional laminar high WSS, which maintains ECs in a quiescent and cytoprotective state characterized by resistance to inflammation, oxidative stress, and apoptosis [106]. Supra-high, low, or oscillatory WSS promote endothelial dysfunction. Several studies have demonstrated the importance of high levels of WSS together with a positive WSS gradient (i.e., accelerating flow) in the formation of IAs (for reviews, see [15, 71]). Once the aneurysm is formed, flow within the dome is influenced by the location of the aneurysm, the geometry of the aneurysm and of the parental artery, and by the aneurysm neck characteristics. Two types of saccular aneurysms are found in the circle of Willis, in which blood flow patterns are different. The IAs that are the most often observed are located at the bifurcation between two arteries. In this type of IA, the blood flow from the parent artery impinges directly to the dome and moves in the direction of the neck [57]. In the case of sidewall IAs, which are located on the lateral wall of an artery, the blood flow jet impinges first on the neck of the IA before circulating inside the dome [57]. In sidewall IAs, the highest wall tension is expected to be at the level of the neck whereas in IAs located at bifurcation the highest wall tension is supposed to be on the dome.

Throughout the lifetime of an aneurysm, the aneurysmal wall will experience different flow patterns and implications of both low WSS and supra-high WSS have been demonstrated to affect its growth and rupture. Effects of the different WSS patterns on vascular wall changes have been recently reviewed by Staarmann et al. [88]. In brief, low WSS induces disorganization of ECs, decreased production of prostacyclin and tissue plasminogen activator, increased production of vasoconstrictive, inflammatory agents and oxidative stress, and augment EC apoptosis. Supra-high WSS leads to the production of MMPs by ECs and SMCs, to an increase of nuclear factor-kappa B, cyclooxygenase-2, and interleukin-1β, to the migration of the SMCs, to the SMC phenotypic change from contractile to secretory, to the release of platelet-derived growth factor and fibroblast growth factor-2 by SMCs, and to increased production of tissue plasminogen activator. Negative WSS gradient (i.e., decelerating flow) counteracts the upregulation of genes normally induced by physiological WSS. Positive WSS gradient downregulates genes inhibiting cell cycle progression and proliferation, decreases anti-apoptotic, inflammatory and chemotaxis gene expression, and favors ECM degradation. In a recent study performed on rats, Shimizu et al. [83] demonstrated that low WSS and high oscillatory shear index (OSI, i.e., magnitude of WSS fluctuations as a function of cardiac cycle) co-localized with regions of aneurysm growth, and that such regions were highly infiltrated by macrophages. The authors also showed that neutrophils and proliferative SMCs were more abundant in a growing aneurysm in comparison to a stable one, and suggest that this was associated with the upregulation of the cellular communication network factor 1 [82]. Besides a description of all these cellular modifications, the exact mechanism linking biomechanical forces to IA growth and rupture is not yet known.

### Wall shear stress and intracranial aneurysm risk of rupture

**Fig. 3:**
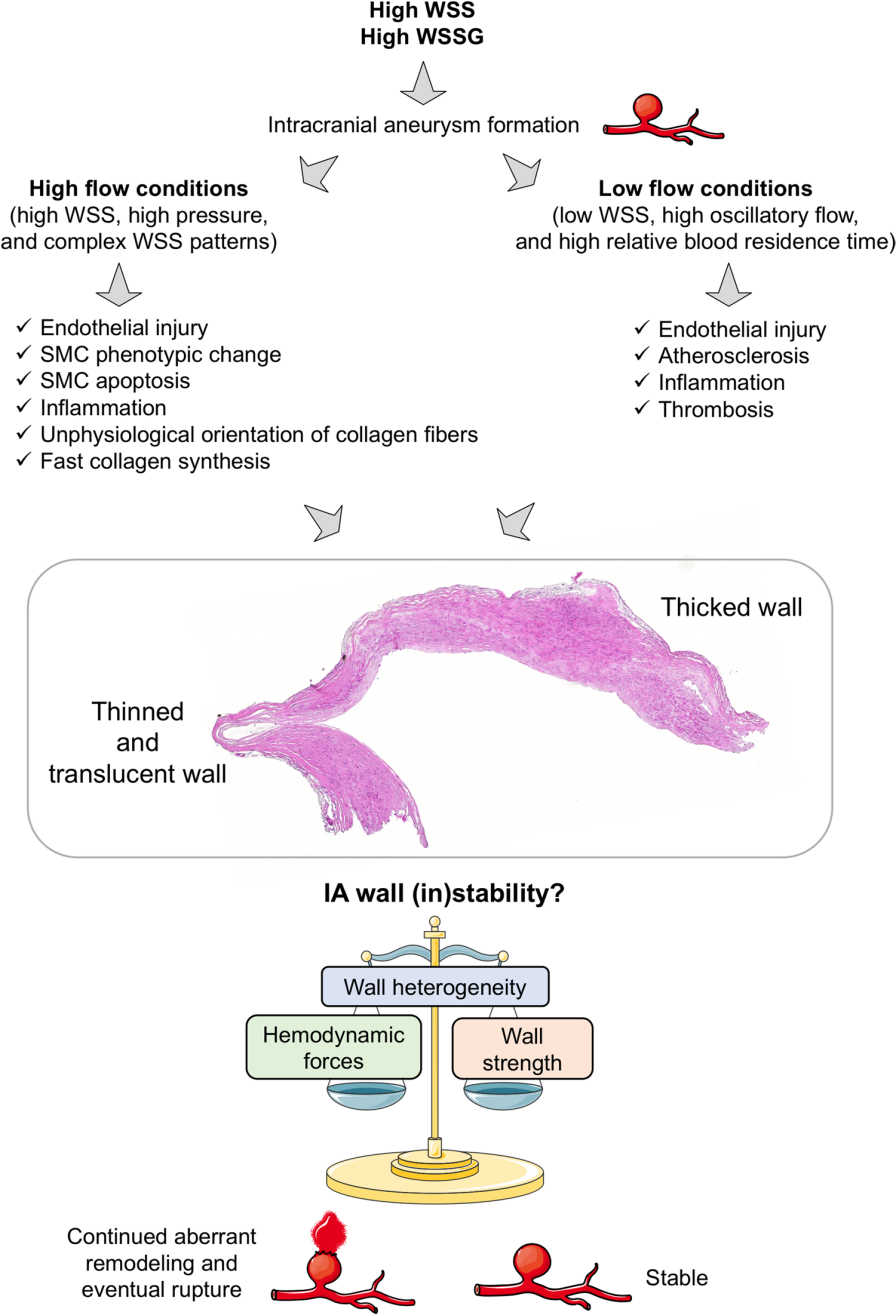
Flow chart of the different hemodynamic factors leading to intracranial aneurysm formation and rupture. High wall shear stress (WSS) and WSS gradient (WSSG) lead to the formation of intracranial aneurysm. Once the IA is formed, wall composition and heterogeneity characterizing IA (in)stability are influenced by the different hemodynamics forces. OSI: oscillatory shear index, SMC: smooth muscle cell. Adapted from [71, 87]

Many studies investigating the association between WSS and the risk of rupture of an IA have been performed by computational fluid dynamics (CFD). Using computed tomography angiography (CTA), magnetic resonance angiography (MRA), 3D digital subtraction angiography (DSA), or 3D digital rotational angiography (DRA), CFD creates 3D models of blood flow. The different studies performed on animal models and in humans give contradictory results about the implication of low or supra-high WSS in IA growth and rupture. Based on histological investigations, intraoperative image analysis, or vessel wall imaging, authors have linked the different conditions of WSS to endothelial dysfunction, SMC turn-over, inflammatory cell infiltration, presence of atherosclerotic plaques, and wall thinning or thickening with sometimes opposite conclusions [71]. However, two mechanistic pathways, one involving low WSS and high OSI, and one involving high WSS, high WSS gradient, and low OSI, have been proposed to explain IA wall remodeling and rupture (Fig. 3) [71, 87]. In all cases, when the wall strength is lower than the hemodynamic forces, the aneurysmal wall ruptures. Further investigations have to be conducted to definitely fix the role of the different patterns of WSS in IA wall remodeling and rupture. Mechanical strength varies from patient to patient, even when comparing only unruptured IAs, and also varies locally within the same IA dome [74]. Once the inner elastic lamina (IEL) is severely damaged or lost, collagen fibers are the main load-bearing structure in the IA wall. Their presence and organization will determine the strength of the aneurysmal wall. Robertson et al. [74] showed that there is a high variability in the collagen fiber diameter and architecture between IAs. Collagen fibers are densely packed in some samples, whereas they have an abnormal sparse structure in others. Moreover, the average diameter of collagen fibers is different between the luminal side of the IA and the medial part suggesting different processes for formation and maintenance. Cebral et al. [9] have shown that collagen fiber diameter is larger when WSS increased and is smaller with increasing OSI. They also observed that in regions with normal WSS (between 5 and 70 dynes/cm^2^) or low WSS (< 5 dynes/cm^2^) collagen fibers had a more coherent structure, i.e., collagen fibers organized in two principal orientations, than in regions exposed to supra-high WSS (> 70 dynes/cm^2^) in which collagen fibers are aligned in only one direction with gaps between the fibers.

### Limitation of computational fluid dynamics use in clinics

CFD analyses brought a lot in the research related to IAs, but they are often based on general boundary conditions. Indeed, most CFD studies assume that arteries and aneurysms have rigid and homogeneous walls, and physiological information such as blood pressure, heart rate, blood viscosity, or flow velocity are fixed conditions for all people. Moreover, CFD analyses need high-quality images which are not always available, and no precise processes have been validated by a consortium of experts. Nowadays, CFD analyses are time consuming and need specific competences which are not always available in hospitals; there is no simple procedure that can be easily carried out by a technician. However, CFD analyses are of high interest in research to better understand the effects of biomechanical forces for the aneurysmal wall properties and (in)stability, mostly if they are carried out in conjunction with detailed histological analyses (see [2] for an example).

### Perspectives

Performing CFD analyses routinely in clinics to evaluate the risk of rupture of an IA is difficult because it requires a specific training and the use of a dedicated software. Currently, as for histological investigations, such type of information cannot yet directly help the clinician in the decision to observe or to treat an unruptured IA. Factors used in routine clinical practice by clinicians are radiological images, and recent advances in this field are promising.

## High-resolution vessel wall magnetic resonance imaging to evaluate intracranial aneurysm wall (in)stability

The traditional techniques used to visualize the lumen of the blood vessels are CTA, MRA, DRA, or DSA. Unruptured IAs are often incidentally detected when one of these radiological exams is prescribed for diagnosis of any other disorder. Radiological imaging techniques regularly improved. High-resolution vessel wall magnetic resonance imaging (HR-VW MRI) after gadolinium administration is an emergent technique providing information about aneurysm wall properties [79]. Presence of contrast agent in the aneurysmal wall can be visualized by double inversion recovery black blood sequence. This technique suppresses signals of the vessel lumen and of the cerebrospinal fluid. This method complements the traditional ones evaluating the lumen of IAs. The use of HR-VW MRI was firstly proposed to identify the site of rupture in case of aneurysmal SAH [51]. Indeed, ruptured IAs demonstrated thick vessel wall enhancement in comparison to unruptured IAs. HR-VW MRI is now proposed to be used to identify unstable IAs prone to rupture based on the presence of aneurysm wall enhancement (AWE). Qualitative and quantitative methods have been described to assess AWE [79]. The simplest method is to discriminate IAs with or without AWE. Other qualitative methods classify AWE as faint/strong [58], focal/circumferential [17, 23], or thin/thick [17]. Nagahata et al. [58] defined “strong AWE” as aneurysm enhancement equal to the one in choroid plexus or venous plexus, and “faint AWE” as an increased wall signal intensity from pre- to post-contrast scan. In 2018, Edjlali et al. [17] proposed a 4-grade classification: grade 0 = no or questionable focal AWE; grade 1 = focal thick (> 1 mm) AWE; grade 2 = thin (≤ 1 mm) circumferential AWE; and grade 3 = thick (> 1 mm) circumferential AWE. Quantitative methods for AWE evaluation are based on the measurement of the wall enhancement index defined as the change in IA wall signal intensity before and after gadolinium injection, or on the measurement of the aneurysm-to-pituitary stalk ratio [79].

### Wall enhancement and intracranial aneurysm characteristics

Presence of AWE has been correlated to anatomical characteristics of IAs such as size, location, and shape [52, 65, 70, 73, 76, 100, 108, 109]. AWE is described to be higher in larger IAs [65, 73, 76]. However, AWE can also be observed in small IAs (size < 7 mm) [76]. High depth/neck width aspect ratio is shown to be associated with higher AWE [73, 100]. Larger AWE values are more often obtained from IAs located in anterior cerebral artery, posterior communicating artery, posterior circulation artery, or middle cerebral artery [76]. Some of these locations are considered at high risk of rupture in the scores used in clinics (PHASES, UIAT, and ELAPSS scores). Irregular IA shape can lead to a change in the blood flow pattern associated with endothelial dysfunction leading to increased permeability or alternatively to stagnation of contrast agent. Although this is not always the case, higher AWE is often observed in presence of IA irregular shape [52, 70, 76, 100, 108, 109]. Presence of a daughter sac has been associated with heterogeneous AWE, with AWE present in the main aneurysmal sac but often absent from the daughter sac [52].

AWE has been shown to co-localize with low time-average WSS, low maximum OSI, and a large low shear area [42, 47, 104, 107], suggesting that AWE is associated with low WSS conditions. Further investigations towards the links between hemodynamics and AWE are warranted. Some studies have investigated whether AWE correlates with scores used in clinics to evaluate the risk of rupture of a specific IA. Positive correlations have been observed between AWE and PHASES score [33, 47, 65, 73, 108], UIAT score [65], or ELAPSS score [73, 108]. However, absence of correlation between UIAT score and AWE has also been shown [73]. Hartman et al. [33] described that IAs with a PHASES score higher than 3 present more often wall thinning and AWE. So far, no association between AWE and smoking status, the daily use of acetylsalicylic acid or statins, hypertension, sex, diabetes, or family history of IAs have been shown [73, 76, 108, 109].

### Wall enhancement and intracranial aneurysm wall composition

AWE was firstly used to identify the culprit IA in case of aneurysmal SAH [51]. Indeed, almost all ruptured IAs present wall enhancement on imaging. In such IAs, based on intraoperative inspections and/or histological investigations, AWE has been associated with inflammatory cells infiltration [36, 58] or presence of hemostatic thrombus at the rupture site [53]. More particularly, it has been observed that circumferential AWE was more associated with abundant inflammation and neovascularization whereas focal AWE seems to be more observed in the case of fresh intraluminal thrombus retaining contrast agent [53]. As inflammation was also described in unruptured IAs [21, 22, 41, 55] and proposed to predispose to IA instability, it has been suggested to use absence/presence of AWE to discriminate between stable and unstable unruptured IAs. Thus, positive correlation between AWE and presence of neutrophils or macrophages inside the wall of unruptured IAs has been shown [48, 70, 85, 109]. In aneurysms exhibiting AWE but no myeloperoxidase activity, presence of *vasa* *vasorum* was described [48]. *Vasa* *vasorum* are normally absent in non-diseased intracranial arteries, but they can develop in the context of hypoxia in atherosclerotic lesions and wall remodeling. Thereby, AWE was observed when atherosclerotic lesions and neovascularization were present in the aneurysmal wall [70, 85, 109]. Moreover, atherosclerotic plaques in unruptured IAs seem to be more associated with focal AWE than with uniform AWE [70]. By comparing the concentration of blood lipoprotein(a) between the lumen of unruptured IA and the parental artery, Ishii et al. [37] have shown that a higher lipoprotein(a) concentration was associated with increased AWE in unruptured IAs. AWE is also higher in thrombosed aneurysmal walls [100, 109]. Finally, Matsushige et al. [52] have reported that focal and circumferential AWE are observed in thin (20–50 µm) and thick (120–320 µ m) walls, respectively. Altogether, studies performed on unruptured IAs show that AWE could be due to wall thickening with inflammatory cell infiltration and presence of *vasa* *vasorum*, wall thinning with compromised endothelial barrier integrity, or intramural hematoma.

### Wall enhancement used in clinical practice

With respect to ruptured IAs, AWE can be used to identify the culprit IA in patients with multiple IAs. Based on the pattern of AWE, the identification of an intraluminal thrombus (focal AWE), suggestive for the site of IA dome rupture, could help in clinical management [54]. Currently, no consensus exists onto the use of HR-VW MRI to detect and interpret AWE in unruptured IAs in daily clinical practice. However, whatever the method used to classify absence/presence of AWE or grading of AWE, the different investigators seem to be able to discriminate stable and unstable IAs using HR-VW MRI. A recent systematic review and meta-analysis performed on more than 500 aneurysms coming from 6 studies has shown that the sensitivity of AWE to screen unstable IAs is high (95%) [92]. Importantly, the authors also showed that the absence of AWE is strongly associated with IA stability (negative predictive value of 96%). In a prospective cohort of 145 unruptured small IAs followed over 2 years, Gariel et al. [25]. have demonstrated that increased AWE was a marker of IA wall growth and instability. In two retrospective longitudinal studies, the authors showed that AWE was more frequent in IAs with morphological changes than in stable IAs [52, 97]. Longer longitudinal follow-up studies are needed to confirm the use of AWE as an independent biomarker of wall instability. Vessel wall permeability can be evaluated by dynamic contrast-enhanced MRI (DCE MRI). Using this technique, presence of leaky regions in the aneurysmal wall has been shown [69], and comparison with AWE imaging showed that 66% of the aneurysms having leaky regions had also noticeable AWE. However, 50% of the IAs with AWE did not show leakage. Conversely, leakage was also observed in IAs without AWE [69]. This data support the idea that the combination of HR-VW MRI and DCE MRI may provide additional information about aneurysm wall vulnerability.

### Other imaging modalities to determine intracranial aneurysm wall instability

Another type of imaging using ferumoxytol or MPO-gadolinium has been proposed to characterize unstable inflamed IAs. Ferumoxytol is a superparamagnetic particle of iron oxide phagocyted by macrophages. Ferumoxytol uptake inside the aneurysmal wall during the first 24 h post-infusion was shown predictive for a high risk of rupture during the 6 following months [34]. However, because of its potential risk of lethal allergic reaction, the use of ferumoxytol allowed by the Food and Drug Administration is strictly limited to iron deficiency anemia in the context of chronic kidney disease. In addition, macrophage imaging by ferumoxytol in the aneurysmal wall is technically challenging and time consuming, limiting its use in the context of aneurysmal disease. Recently, the use of chelated MPO-gadolinium on histological sections of human IA in ultra-high resolution 7 T MRI has demonstrated the presence of neutrophil activity in the aneurysmal wall [99]. This study suggests that agents targeted at immunological responses are possible in cerebral aneurysms and may provide useful additional information.

### Perspectives

High-resolution vessel wall imaging is a very promising tool in the context of IA disease, and would certainly be of great help to better characterize IA wall (in)stability and help clinicians in their decision to observe or treat an unruptured IA. Some technologies described in this section are still at their infancy; development of new cell-specific contrast agents and sensors is of high interest and would help to better discriminate between stable and unstable IAs. One of the remaining challenges is to be able to measure in vivo precisely aneurysmal wall thickness which is at the moment beyond the resolution of conventional imaging methods used in clinics.

## Common data elements to improve the current state of knowledge

Until recently, clinical and fundamental research in the field of unruptured IAs and SAH suffered from the lack of standardized definitions. In 2014, the National Institute of Neurological Disorders and Stroke, in collaboration with the Neurocritical Care Society and the National Library of Medicine, initiated the Common Data Elements (CDEs) project for unruptured IAs and SAH. The goal of this project was to elaborate common definitions concerning IA and SAH, and to create data sets to capture and record information consistently across hospitals and research teams. In 2019, 8 articles describing more than 1300 CDEs about unruptured IAs and SAH have been published to define (1) subject characteristics [5], (2) assessments and examinations [12], (3) hospital course and acute therapies [13], (4) biospecimens and biomarkers [11], (5) imaging [30], (6) long-term therapies [103], (7) cohort studies and clinical trials [29], and (8) outcomes and endpoints [90]. The process of selection of each CDE was based on CDEs previously defined in the field of neurovascular diseases, on literature, on experience of group members and experts, on observational studies, and on clinical trials. CDEs have been classified as disease core CDEs (essential information applicable to any unruptured IAs and SAH research study), supplemental-highly recommended CDEs (essential information based on certain conditions or study types), supplemental CDEs (information commonly collected but whose relevance depends on study design or research type), and exploratory CDEs (information requiring validation). The core CDEs defined by the different working groups are presented in Table 8. CDEs are valuable resources for researchers; they will facilitate clinical research and clinical trials, data sharing, and data analyses. For the next years, they should encourage communication and progress in the field of unruptured IAs and SAH. CDEs will participate in the improvement of the understanding of the disease and will provide additional information to determine whether an unruptured IA is at risk of rupture or not. The core CDEs would certainly be valuable extra information to add to the current clinical scores used to decide whether an unruptured IA has to be treated or not.

**Table 8:**
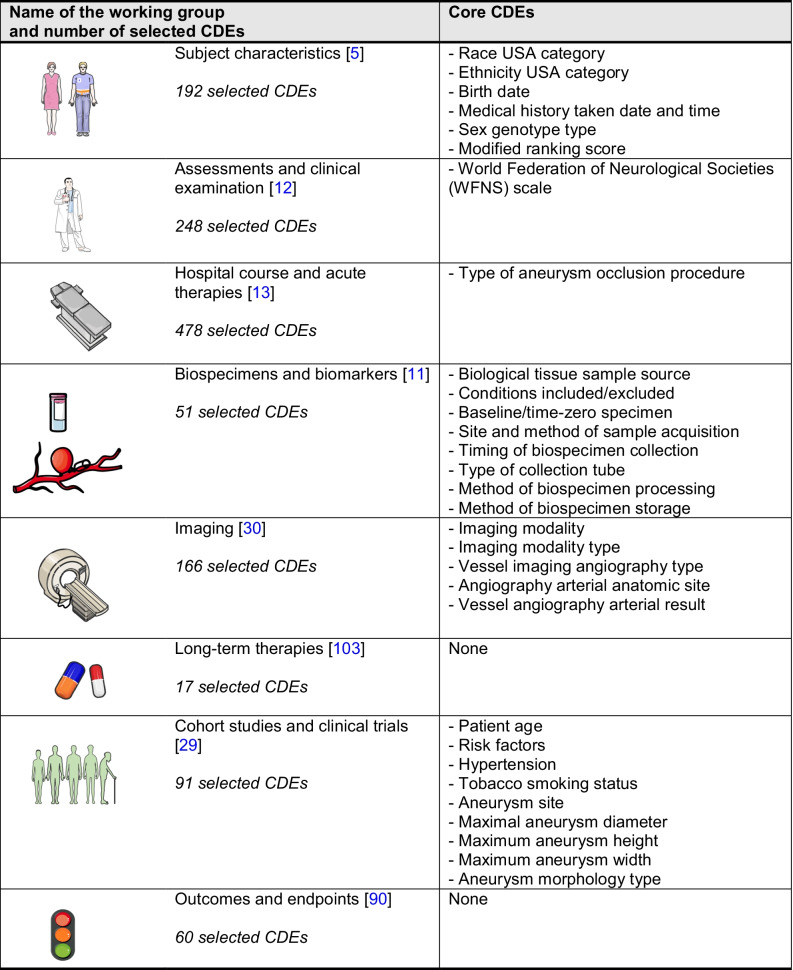
Core CDEs defined by the 8 working groups related to the CDEs project for unruptured IAs and SAH

## Genetics in intracranial aneurysm susceptibility

Increasing evidence suggest a genetic component in IA formation and predisposition to rupture. Heritable conditions such as autosomal dominant PKD, Ehlers-Danlos syndrome, Loeys-Dietz syndrome, Marfan syndrome, neurofibromatosis type I, or hereditary hemorrhagic telangiectasia have an IA prevalence of 4–40%, 12–17.5%, 10–28%, 0–14%, 9–11%, and 10%, respectively, which is higher than the prevalence described for the general population (for a review, see [105]). Japanese or Finnish people present a higher risk for IA rupture supporting the idea of possible genetic influence. Familial cases represent 10% of all diagnosed IAs. Patients with a familial history of IA seem to be more likely to develop IAs, more particularly multiple IAs, and they present a higher risk of rupture with a poorer outcome after the rupture. Moreover, patient’s age and IA size at the rupture tend to be lower in such families. Several groups have investigated the genetic component of IA formation and risk of rupture in familial and non-familial cohorts of patients. Thus, genome-wide association studies (GWAS), subsequent case–control replication analyses, and whole-exome sequencing approaches have revealed genes associated with IA susceptibility and have shown that some act in concert with environmental risk factors (for a review, see [105]). In a recent GWAS meta-analysis performed on 10.754 cases and 306.882 controls, 6 previously identified risk loci were confirmed and 11 novel risk loci were identified [3]. Many of these genes have known or putative roles in arterial function and blood pressure regulation. Moreover, gene-mapping and heritability enrichment methods suggest a determining role for ECs in IA development. A high level of similarity in the common variant genetic architecture of unruptured and ruptured IAs was shown. Finally, this study identified blood pressure and smoking, two well-known clinical risk factors for IA, as the main genetic drivers of IA disease.

The variety of genes found in the different studies suggests that multiple pathophysiological pathways are involved in IA formation and/or rupture. The discovery of genes associated with higher risk of IA rupture would allow for the generation of genetically modified animals to better understand the evolution of this disease. Future studies are needed to determine whether the polygenic risk of IA and clinical risk factors are independent risk factors for IA. Such information will certainly contribute to the design of new and improved scores to predict aneurysmal wall in(stability) in clinical practice.

## Future directions

An increasing number of investigators try to develop computational models integrating IA morphology characteristics, hemodynamics, and wall imaging, with the aim to develop IA rupture prediction models. Indeed, mathematical models are under development to simulate the adaptation of the arterial wall to mechanical stress considering arterial wall composition in cells and ECM. As an example, Watton et al. [102] have developed a mathematical growth and remodeling model for saccular IAs in which they included the geometry, the elasticity, and the thickness of the wall as well as elastin and collagen fiber content and orientation, arterial pressure variables, and shear stress parameters. Given that histological and in vitro studies showed that EC and SMC content and function change during IA lifetime, the differential properties of these cells should be added to these mathematical models to generate a dynamic model of the disease.

Machine learning models are progressively being implemented for the detection of IAs on angiographic images, or to discriminate ruptured from unruptured IAs based on morphological parameters, hemodynamics, and patient-related information. Several statistical models have been proposed, but some are only based on small sample size, have not been externally validated, or consider generic boundary conditions for hemodynamic data. Based on a large patient cohort, Detmer et al. [14] have recently developed a logistic regression model for rupture status prediction including various risk factors such as gender, age, number of IAs or IA location, and flow data. This model has been internally and externally validated and gave predictive performances at equivalent or even better than those of other previously published machine learning classifiers. In the future, such models could be further improved by incorporating information about AWE.

For several diseases such as myocardial infarction or brain hemorrhage, endogenous circulating blood molecules have been used as diagnostic markers. Tutino et al. [95] have shown that patients with unruptured IAs present a different RNA profile in circulating neutrophils in comparison to patients without IA. Based on this circulating neutrophil transcriptome data, they developed machine-learning methods able to discriminate patients with or without unruptured IAs with an accuracy ranging from 0.6 to 0.9. More recently, they developed a machine learning classifier derived from whole blood transcriptomes [66]. The crucial role of neutrophils in IA wall degeneration and rupture has been recently demonstrated in rats [43]. Accumulation of neutrophils and production of MMP9 at the site of IA rupture has been shown, suggesting that neutrophils may be suitable candidates for diagnostic markers and/or as therapeutic targets. These findings could open the door to the development of blood markers of IA wall (in)stability.

## Conclusion

**Fig. 4:**
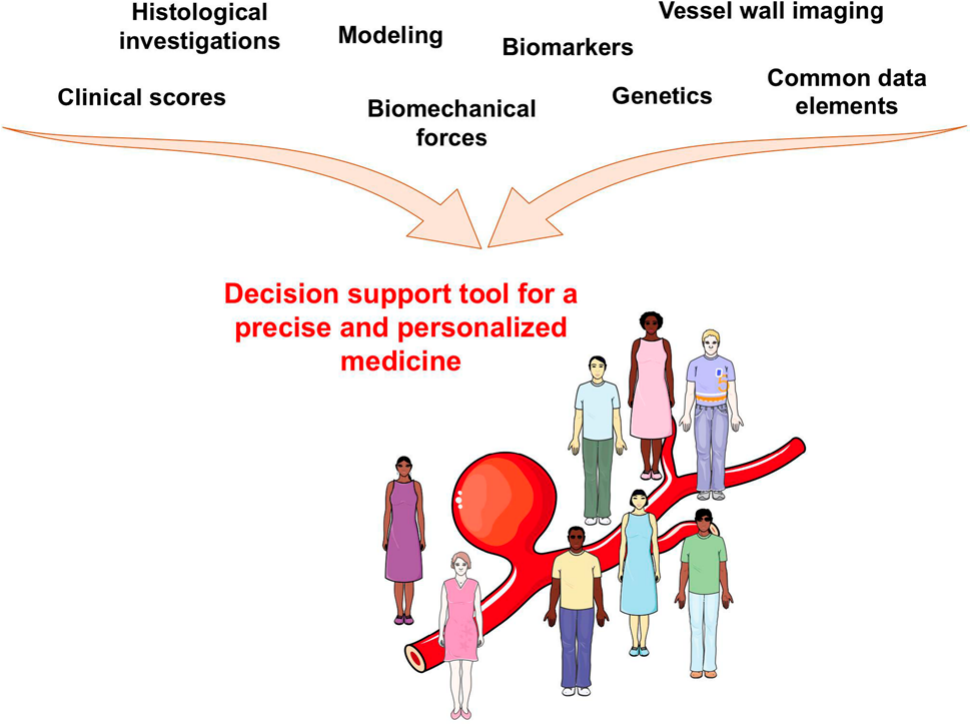
Efforts converging towards a more precise and personalized medicine for patients affected by intracranial aneurysms

Intracranial aneurysm disease, which is observed in 3 to 5% of the population, is a very complex disease whose mechanisms are not totally understood. The main concern with this disease is the difficulty to predict its evolution. Although a large part of the unruptured IAs would never give any symptoms, a rupture will have dramatic consequences as severe handicap or death. As different processes are involved in the wall of evolving IAs, it is currently impossible to safely propose to patients a pharmacological treatment to prevent growth or rupture of their IA. Remodeling of the aneurysmal wall leading to growth and rupture has been characterized by histologic analyses performed in human samples or in animal models. Such studies have shown that rupture of an IA is driven by remodeling of the aneurysmal wall characterized by inflammatory cells infiltration, SMC death, ECM degradation, calcification, or lipid accumulation. Wall remodeling is driven by risk factors such as IA morphology characteristics, smoking, hypertension, sex, and also by hemodynamic forces. Improved understanding of the processes involved in wall remodeling is needed to better define wall (in)stability. Recent advances in vessel wall imaging bring new tools and information to answer this point. The recent creation of the CDEs for IA and SAH will also help clinicians and researchers to better apply standardization to the disease with the final goal to secure their decision to treat or to observe an unruptured IA. Finally, new insights in genetic profiles for IA rupture and circulating biomarkers, and development of predictive models for rupture risk assessment will beyond doubt help to better define which unruptured IA is at risk of rupture and should be treated. Efforts converge towards a more precise and personalized medicine for patients affected by IAs (Fig. 4), which is critical for a better management of the disease.

## Data Availability

Data and pictures shown in the manuscript will be available on demand.

## References

[1] Backes D, Rinkel GJE, Greving JP, Velthuis BK, Murayama Y, Takao H, Ishibashi T, Igase M, terBrugge KG, Agid R, Jaaskelainen JE, Lindgren AE, Koivisto T, von Und Zu, Fraunberg M, Matsubara S, Moroi J, Wong GKC, Abrigo JM, Igase K, Matsumoto K, Wermer MJH, van Walderveen MAA, Algra A, Vergouwen MDI (2017). ELAPSS score for prediction of risk of growth of unruptured intracranial aneurysms. Neurology.

[2] Baeriswyl DC, Prionisti I, Peach T, Tsolkas G, Chooi KY, Vardakis J, Morel S, Diagbouga MR, Bijlenga P, Cuhlmann S, Evans P, Kwak BR, Ventikos Y, Krams R (2019). Disturbed flow induces a sustained, stochastic NF-kappaB activation which may support intracranial aneurysm growth in vivo. Sci Rep.

[3] Bakker MK, van der Spek RAA, van Rheenen W, Morel S, Bourcier R, Hostettler IC, Alg VS, van Eijk KR, Koido M, Akiyama M, Terao C, Matsuda K, Walters RG, Lin K, Li L, Millwood IY, Chen Z, Rouleau GA, Zhou S, Rannikmae K, Sudlow CLM, Houlden H, van den Berg LH, Dina C, Naggara O, Gentric JC, Shotar E, Eugene F, Desal H, Winsvold BS, Borte S, Johnsen MB, Brumpton BM, Sandvei MS, Willer CJ, Hveem K, Zwart JA, Verschuren WMM, Friedrich CM, Hirsch S, Schilling S, Dauvillier J, Martin O, Stroke HA-I, China Kadoorie Biobank Collaborative G, BioBank Japan Project C, Group IS, Group C, Genetics, Observational Subarachnoid Haemorrhage Study i, International Stroke Genetics C, Jones GT, Bown MJ, Ko NU, Kim H, Coleman JRI, Breen G, Zaroff JG, Klijn CJM, Malik R, Dichgans M, Sargurupremraj M, Tatlisumak T, Amouyel P, Debette S, Rinkel GJE, Worrall BB, Pera J, Slowik A, Gaal-Paavola EI, Niemela M, Jaaskelainen JE, von Und Zu Fraunberg M, Lindgren A, Broderick JP, Werring DJ, Woo D, Redon R, Bijlenga P, Kamatani Y, Veldink JH, Ruigrok YM (2020) Genome-wide association study of intracranial aneurysms identifies 17 risk loci and genetic overlap with clinical risk factors. Nat Genet 52:1303-1313. 10.1038/s41588-020-00725-7 10.1038/s41588-020-00725-7 PMC7116530 33199917

[4] Bijlenga P, Gondar R, Schilling S, Morel S, Hirsch S, Cuony J, Corniola MV, Perren F, Rufenacht D, Schaller K (2017). PHASES score for the management of intracranial aneurysm: a cross-sectional population-based retrospective study. Stroke.

[5] Bijlenga P, Morita A, Ko NU, Mocco J, Morel S, Murayama Y, Wermer MJH, Brown RD, Unruptured Cerebral A, Investigators SCP (2019). Common data elements for subarachnoid hemorrhage and unruptured intracranial aneurysms: recommendations from the working group on subject characteristics. Neurocrit Care.

[6] Bjorkman J, Frosen J, Tahtinen O, Huttunen T, Huttunen J, Kurki MI, von Und Zu, Fraunberg M, Koivisto T, Manninen H, Jaaskelainen JE, Lindgren AE (2019). Aneurysm size is the strongest risk factor for intracranial aneurysm growth in the Eastern Finnish population. Neurosurgery.

[7] Brinjikji W, Pereira VM, Khumtong R, Kostensky A, Tymianski M, Krings T, Radovanovich I (2018). PHASES and ELAPSS scores are associated with aneurysm growth: a study of 431 unruptured intracranial aneurysms. World Neurosurg.

[8] Cagnazzo F, Gambacciani C, Morganti R, Perrini P (2017). Intracranial aneurysms in patients with autosomal dominant polycystic kidney disease: prevalence, risk of rupture, and management. A systematic review Acta Neurochir (Wien).

[9] Cebral JR, Duan X, Gade PS, Chung BJ, Mut F, Aziz K, Robertson AM (2016). Regional Mapping of Flow and Wall Characteristics of Intracranial Aneurysms. Ann Biomed Eng.

[10] Chalouhi N, Ali MS, Starke RM, Jabbour PM, Tjoumakaris SI, Gonzalez LF, Rosenwasser RH, Koch WJ, Dumont AS (2012). Cigarette smoke and inflammation: role in cerebral aneurysm formation and rupture. Mediators Inflamm.

[11] Chou SH, Macdonald RL, Keller E, Unruptured Intracranial A, Investigators SCP (2019). Biospecimens and molecular and cellular biomarkers in aneurysmal subarachnoid hemorrhage studies: common data elements and standard reporting recommendations. Neurocrit Care.

[12] Damani R, Mayer S, Dhar R, Martin RH, Nyquist P, Olson DM, Mejia-Mantilla JH, Muehlschlegel S, Jauch EC, Mocco J, Mutoh T, Suarez JI, Unruptured Intracranial A, Investigators SCP (2019). Common data element for unruptured intracranial aneurysm and subarachnoid hemorrhage: recommendations from assessments and clinical examination workgroup/subcommittee. Neurocrit Care.

[13] de Oliveira Manoel AL, van der Jagt M, Amin-Hanjani S, Bambakidis NC, Brophy GM, Bulsara K, Claassen J, Connolly ES, Hoffer SA, Hoh BL, Holloway RG, Kelly AG, Mayer SA, Nakaji P, Rabinstein AA, Vajkoczy P, Vergouwen MDI, Woo H, Zipfel GJ, Suarez JI, Unruptured A, Investigators SCP (2019). Common data elements for unruptured intracranial aneurysms and aneurysmal subarachnoid hemorrhage: recommendations from the working group on hospital course and acute therapies-proposal of a multidisciplinary research group. Neurocrit Care.

[14] Detmer FJ, Luckehe D, Mut F, Slawski M, Hirsch S, Bijlenga P, von Voigt G, Cebral JR (2020). Comparison of statistical learning approaches for cerebral aneurysm rupture assessment. Int J Comput Assist Radiol Surg.

[15] Diagbouga MR, Morel S, Bijlenga P, Kwak BR (2018). Role of hemodynamics in initiation/growth of intracranial aneurysms. Eur J Clin Invest.

[16] Diagbouga MR, Morel S, Cayron AF, Haemmerli J, Georges M, Hierck BP, Allemann E, Lemeille S, Bijlenga P, Kwak BR (2021). Primary cilia control endothelial permeability by regulating expression and location of junction proteins. Cardiovasc Res.

[17] Edjlali M, Guedon A, Ben Hassen W, Boulouis G, Benzakoun J, Rodriguez-Regent C, Trystram D, Nataf F, Meder JF, Turski P, Oppenheim C, Naggara O (2018). Circumferential thick enhancement at vessel wall MRI has high specificity for intracranial aneurysm instability. Radiology.

[18] Etminan N, Brown RD, Beseoglu K, Juvela S, Raymond J, Morita A, Torner JC, Derdeyn CP, Raabe A, Mocco J, Korja M, Abdulazim A, Amin-Hanjani S, Al-Shahi Salman R, Barrow DL, Bederson J, Bonafe A, Dumont AS, Fiorella DJ, Gruber A, Hankey GJ, Hasan DM, Hoh BL, Jabbour P, Kasuya H, Kelly ME, Kirkpatrick PJ, Knuckey N, Koivisto T, Krings T, Lawton MT, Marotta TR, Mayer SA, Mee E, Pereira VM, Molyneux A, Morgan MK, Mori K, Murayama Y, Nagahiro S, Nakayama N, Niemela M, Ogilvy CS, Pierot L, Rabinstein AA, Roos YB, Rinne J, Rosenwasser RH, Ronkainen A, Schaller K, Seifert V, Solomon RA, Spears J, Steiger HJ, Vergouwen MD, Wanke I, Wermer MJ, Wong GK, Wong JH, Zipfel GJ, Connolly ES, Steinmetz H, Lanzino G, Pasqualin A, Rufenacht D, Vajkoczy P, McDougall C, Hanggi D, LeRoux P, Rinkel GJ, Macdonald RL (2015). The unruptured intracranial aneurysm treatment score: a multidisciplinary consensus. Neurology.

[19] Etminan N, Rinkel GJ (2017). Unruptured intracranial aneurysms: development, rupture and preventive management. Nat Rev Neurol.

[20] Foreman PM, Hendrix P, Harrigan MR, Fisher WS, Vyas NA, Lipsky RH, Walters BC, Tubbs RS, Shoja MM, Griessenauer CJ (2018). PHASES score applied to a prospective cohort of aneurysmal subarachnoid hemorrhage patients. J Clin Neurosci.

[21] Frosen J, Piippo A, Paetau A, Kangasniemi M, Niemela M, Hernesniemi J, Jaaskelainen J (2004). Remodeling of saccular cerebral artery aneurysm wall is associated with rupture: histological analysis of 24 unruptured and 42 ruptured cases. Stroke.

[22] Frosen J, Tulamo R, Paetau A, Laaksamo E, Korja M, Laakso A, Niemela M, Hernesniemi J (2012). Saccular intracranial aneurysm: pathology and mechanisms. Acta Neuropathol.

[23] Fu Q, Guan S, Liu C, Wang K, Cheng J (2018). Clinical Significance of Circumferential Aneurysmal Wall Enhancement in symptomatic patients with unruptured intracranial aneurysms: a high-resolution MRI study. Clin Neuroradiol.

[24] Gade PS, Tulamo R, Lee KW, Mut F, Ollikainen E, Chuang CY, Jae Chung B, Niemela M, Rezai Jahromi B, Aziz K, Yu A, Charbel FT, Amin-Hanjani S, Frosen J, Cebral JR, Robertson AM (2019). Calcification in human intracranial aneurysms is highly prevalent and displays both atherosclerotic and nonatherosclerotic types. Arterioscler Thromb Vasc Biol.

[25] Gariel F, Ben Hassen W, Boulouis G, Bourcier R, Trystram D, Legrand L, Rodriguez-Regent C, Saloner D, Oppenheim C, Naggara O, Edjlali M (2020). Increased Wall Enhancement During Follow-Up as a Predictor of Subsequent Aneurysmal Growth. Stroke.

[26] Gerzanich V, Zhang F, West GA, Simard JM (2001). Chronic nicotine alters NO signaling of Ca(2+) channels in cerebral arterioles. Circ Res.

[27] Gondar R, Gautschi OP, Cuony J, Perren F, Jagersberg M, Corniola MV, Schatlo B, Molliqaj G, Morel S, Kulcsar Z, Mendes Pereira V, Rufenacht D, Schaller K, Bijlenga P (2016). Unruptured intracranial aneurysm follow-up and treatment after morphological change is safe: observational study and systematic review. J Neurol Neurosurg Psychiatry.

[28] Greving JP, Wermer MJ, Brown RD, Morita A, Juvela S, Yonekura M, Ishibashi T, Torner JC, Nakayama T, Rinkel GJ, Algra A (2014). Development of the PHASES score for prediction of risk of rupture of intracranial aneurysms: a pooled analysis of six prospective cohort studies. Lancet Neurol.

[29] Hackenberg KAM, Algra A, Salman RA, Frosen J, Hasan D, Juvela S, Langer D, Meyers P, Morita A, Rinkel G, Etminan N, Unruptured A, Investigators SCP (2019). Definition and prioritization of data elements for cohort studies and clinical trials on patients with unruptured intracranial aneurysms: proposal of a multidisciplinary research group. Neurocrit Care.

[30] Hackenberg KAM, Etminan N, Wintermark M, Meyers PM, Lanzino G, Rufenacht D, Krings T, Huston J, Rinkel G, Derdeyn C, Unruptured Intracranial A, Investigators SCP (2019). Common data elements for radiological imaging of patients with subarachnoid hemorrhage: proposal of a multidisciplinary research group. Neurocrit Care.

[31] Hackenberg KAM, Rajabzadeh-Oghaz H, Dreier R, Buchholz BA, Navid A, Rocke DM, Abdulazim A, Hanggi D, Siddiqui A, Macdonald RL, Meng H, Etminan N (2020) Collagen turnover in relation to risk factors and hemodynamics in human intracranial aneurysms. Stroke:STROKEAHA120029335. 10.1161/STROKEAHA.120.029335 10.1161/STROKEAHA.120.029335 PMC7340076 32192404

[32] Hallikainen J, Lindgren A, Savolainen J, Selander T, Jula A, Narhi M, Koivisto T, Kellokoski J, Ylostalo P, Suominen AL, Frosen J (2020). Periodontitis and gingival bleeding associate with intracranial aneurysms and risk of aneurysmal subarachnoid hemorrhage. Neurosurg Rev.

[33] Hartman JB, Watase H, Sun J, Hippe DS, Kim L, Levitt M, Sekhar L, Balu N, Hatsukami T, Yuan C, Mossa-Basha M (2019). Intracranial aneurysms at higher clinical risk for rupture demonstrate increased wall enhancement and thinning on multicontrast 3D vessel wall MRI. Br J Radiol.

[34] Hasan D, Chalouhi N, Jabbour P, Dumont AS, Kung DK, Magnotta VA, Young WL, Hashimoto T, Winn HR, Heistad D (2012). Early change in ferumoxytol-enhanced magnetic resonance imaging signal suggests unstable human cerebral aneurysm: a pilot study. Stroke.

[35] Hernandez-Duran S, Mielke D, Rohde V, Malinova V (2018). The application of the unruptured intracranial aneurysm treatment score: a retrospective, single-center study. Neurosurg Rev.

[36] Hu P, Yang Q, Wang DD, Guan SC, Zhang HQ (2016). Wall enhancement on high-resolution magnetic resonance imaging may predict an unsteady state of an intracranial saccular aneurysm. Neuroradiology.

[37] Ishii D, Zanaty M, Roa JA, Li L, Lu Y, Sabotin R, Allan L, Samaniego EA, Hasan DM (2021). Concentration of Lp(a) (Lipoprotein[a]) in aneurysm sac is associated with wall enhancement of unruptured intracranial aneurysm. Stroke.

[38] Juchler N, Schilling S, Bijlenga P, Morel S, Rufenacht D, Kurtcuoglu V, Hirsch S (2020). Shape irregularity of the intracranial aneurysm lumen exhibits diagnostic value. Acta Neurochir (Wien).

[39] Jufri NF, Mohamedali A, Avolio A, Baker MS (2015). Mechanical stretch: physiological and pathological implications for human vascular endothelial cells. Vasc Cell.

[40] Kadasi LM, Dent WC, Malek AM (2013). Cerebral aneurysm wall thickness analysis using intraoperative microscopy: effect of size and gender on thin translucent regions. J Neurointerv Surg.

[41] Kataoka K, Taneda M, Asai T, Kinoshita A, Ito M, Kuroda R (1999). Structural fragility and inflammatory response of ruptured cerebral aneurysms. A comparative study between ruptured and unruptured cerebral aneurysms. Stroke.

[42] Khan MO, Toro Arana V, Rubbert C, Cornelius JF, Fischer I, Bostelmann R, Mijderwijk HJ, Turowski B, Steiger HJ, May R, Petridis AK (2020) Association between aneurysm hemodynamics and wall enhancement on 3D vessel wall MRI. J Neurosurg:1–11. 10.3171/2019.10.JNS191251 10.3171/2019.10.JNS191251 31923894

[43] Kushamae M, Miyata H, Shirai M, Shimizu K, Oka M, Koseki H, Abekura Y, Ono I, Nozaki K, Mizutani T, Aoki T (2020). Involvement of neutrophils in machineries underlying the rupture of intracranial aneurysms in rats. Sci Rep.

[44] Laaksamo E, Ramachandran M, Frosen J, Tulamo R, Baumann M, Friedlander RM, Harbaugh RE, Hernesniemi J, Niemela M, Raghavan ML, Laakso A (2012). Intracellular signaling pathways and size, shape, and rupture history of human intracranial aneurysms. Neurosurgery.

[45] Laaksamo E, Tulamo R, Baumann M, Dashti R, Hernesniemi J, Juvela S, Niemela M, Laakso A (2008). Involvement of mitogen-activated protein kinase signaling in growth and rupture of human intracranial aneurysms. Stroke.

[46] Laaksamo E, Tulamo R, Liiman A, Baumann M, Friedlander RM, Hernesniemi J, Kangasniemi M, Niemela M, Laakso A, Frosen J (2013). Oxidative stress is associated with cell death, wall degradation, and increased risk of rupture of the intracranial aneurysm wall. Neurosurgery.

[47] Larsen N, Fluh C, Saalfeld S, Voss S, Hille G, Trick D, Wodarg F, Synowitz M, Jansen O, Berg P (2020). Multimodal validation of focal enhancement in intracranial aneurysms as a surrogate marker for aneurysm instability. Neuroradiology.

[48] Larsen N, von der Brelie C, Trick D, Riedel CH, Lindner T, Madjidyar J, Jansen O, Synowitz M, Fluh C (2018). Vessel wall enhancement in unruptured intracranial aneurysms: an indicator for higher risk of rupture? High-resolution MR imaging and correlated histologic findings. AJNR Am J Neuroradiol.

[49] Lawton MT, Vates GE (2017). Subarachnoid hemorrhage. N Engl J Med.

[50] Liu P, Song Y, Zhou Y, Liu Y, Qiu T, An Q, Song J, Li P, Shi Y, Li S, Quan K, Yang GY, Zhu W (2018). Cyclic mechanical stretch induced smooth muscle cell changes in cerebral aneurysm progress by reducing collagen type IV and collagen type VI levels. Cell Physiol Biochem.

[51] Matouk CC, Mandell DM, Gunel M, Bulsara KR, Malhotra A, Hebert R, Johnson MH, Mikulis DJ, Minja FJ (2013). Vessel wall magnetic resonance imaging identifies the site of rupture in patients with multiple intracranial aneurysms: proof of principle. Neurosurgery.

[52] Matsushige T, Shimonaga K, Ishii D, Sakamoto S, Hosogai M, Hashimoto Y, Kaneko M, Ono C, Mizoue T, Kurisu K (2019). Vessel wall imaging of evolving unruptured intracranial aneurysms. Stroke.

[53] Matsushige T, Shimonaga K, Mizoue T, Hosogai M, Hashimoto Y, Kaneko M, Ono C, Ishii D, Sakamoto S, Kurisu K (2019). Focal aneurysm wall enhancement on magnetic resonance imaging indicates intraluminal thrombus and the rupture point. World Neurosurg.

[54] Matsushige T, Shimonaga K, Mizoue T, Hosogai M, Hashimoto Y, Takahashi H, Kaneko M, Ono C, Ishii D, Sakamoto S, Kurisu K (2019). Lessons from vessel wall imaging of intracranial aneurysms: new era of aneurysm evaluation beyond morphology. Neurol Med Chir (Tokyo).

[55] Morel S, Diagbouga MR, Dupuy N, Sutter E, Braunersreuther V, Pelli G, Corniola M, Gondar R, Jagersberg M, Isidor N, Schaller K, Bochaton-Piallat ML, Bijlenga P, Kwak BR (2018). Correlating clinical risk factors and histological features in ruptured and unruptured human intracranial aneurysms: the Swiss AneuX Study. J Neuropathol Exp Neurol.

[56] Morel S, Karol A, Graf V, Pelli G, Richter H, Sutter E, Braunersreuther V, Frosen J, Bijlenga P, Kwak BR, Nuss KM (2019) Sex-related differences in wall remodeling and intraluminal thrombus resolution in a rat saccular aneurysm model. J Neurosurg:1–14. 10.3171/2019.9.JNS191466 10.3171/2019.9.JNS191466 31881533

[57] Munarriz PM, Gomez PA, Paredes I, Castano-Leon AM, Cepeda S, Lagares A (2016). Basic principles of hemodynamics and cerebral aneurysms. World Neurosurg.

[58] Nagahata S, Nagahata M, Obara M, Kondo R, Minagawa N, Sato S, Sato S, Mouri W, Saito S, Kayama T (2016). Wall enhancement of the intracranial aneurysms revealed by magnetic resonance vessel wall imaging using three-dimensional turbo spin-echo sequence with motion-sensitized driven-equilibrium: a sign of ruptured aneurysm?. Clin Neuroradiol.

[59] Oka M, Ono I, Shimizu K, Kushamae M, Miyata H, Kawamata T, Aoki T (2020). The bilateral ovariectomy in a female animal exacerbates the pathogenesis of an intracranial aneurysm. Brain Sci.

[60] Ollikainen E, Tulamo R, Frosen J, Lehti S, Honkanen P, Hernesniemi J, Niemela M, Kovanen PT (2014). Mast cells, neovascularization, and microhemorrhages are associated with saccular intracranial artery aneurysm wall remodeling. J Neuropathol Exp Neurol.

[61] Ollikainen E, Tulamo R, Lehti S, Hernesniemi J, Niemela M, Kovanen PT, Frosen J (2018). Myeloperoxidase associates with degenerative remodeling and rupture of the saccular intracranial aneurysm wall. J Neuropathol Exp Neurol.

[62] Ollikainen E, Tulamo R, Lehti S, Lee-Rueckert M, Hernesniemi J, Niemela M, Yla-Herttuala S, Kovanen PT, Frosen J (2016). Smooth muscle cell foam cell formation, apolipoproteins, and ABCA1 in intracranial aneurysms: implications for lipid accumulation as a promoter of aneurysm wall rupture. J Neuropathol Exp Neurol.

[63] Olsen I, Yamazaki K (2019). Can oral bacteria affect the microbiome of the gut?. J Oral Microbiol.

[64] Pagiola I, Mihalea C, Caroff J, Ikka L, Chalumeau V, Iacobucci M, Ozanne A, Gallas S, Marques M, Nalli D, Carrete H, Caldas JG, Frudit ME, Moret J, Spelle L (2019) The PHASES score: to treat or not to treat? Retrospective evaluation of the risk of rupture of intracranial aneurysms in patients with aneurysmal subarachnoid hemorrhage. J Neuroradiol. 10.1016/j.neurad.2019.06.003 10.1016/j.neurad.2019.06.003 31400432

[65] Petridis AK, Filis A, Chasoglou E, Fischer I, Dibue-Adjei M, Bostelmann R, Steiger HJ, Turowski B, May R (2018). Aneurysm wall enhancement in black blood MRI correlates with aneurysm size. Black blood MRI could serve as an objective criterion of aneurysm stability in near future. Clin Pract.

[66] Poppenberg KE, Li L, Waqas M, Paliwal N, Jiang K, Jarvis JN, Sun Y, Snyder KV, Levy EI, Siddiqui AH, Kolega J, Meng H, Tutino VM (2020). Whole blood transcriptome biomarkers of unruptured intracranial aneurysm. PLoS ONE.

[67] Pyysalo MJ, Pyysalo LM, Pessi T, Karhunen PJ, Lehtimaki T, Oksala N, Ohman JE (2016). Bacterial DNA findings in ruptured and unruptured intracranial aneurysms. Acta Odontol Scand.

[68] Pyysalo MJ, Pyysalo LM, Pessi T, Karhunen PJ, Ohman JE (2013). The connection between ruptured cerebral aneurysms and odontogenic bacteria. J Neurol Neurosurg Psychiatry.

[69] Qi H, Liu X, Liu P, Yuan W, Liu A, Jiang Y, Li Y, Sun J, Chen H (2019). Complementary roles of dynamic contrast-enhanced MR imaging and postcontrast vessel wall imaging in detecting high-risk intracranial aneurysms. AJNR Am J Neuroradiol.

[70] Quan K, Song J, Yang Z, Wang D, An Q, Huang L, Liu P, Li P, Tian Y, Zhou L, Zhu W (2019). Validation of wall enhancement as a new imaging biomarker of unruptured cerebral aneurysm. Stroke.

[71] Rajabzadeh-Oghaz H, Siddiqui AH, Asadollahi A, Kolega J, Tutino VM (2021) The association between hemodynamics and wall characteristics in human intracranial aneurysms: a review. Neurosurg Rev. 10.1007/s10143-021-01554-w 10.1007/s10143-021-01554-w 33913050

[72] Ravindra VM, de Havenon A, Gooldy TC, Scoville J, Guan J, Couldwell WT, Taussky P, MacDonald JD, Schmidt RH, Park MS (2018). Validation of the unruptured intracranial aneurysm treatment score: comparison with real-world cerebrovascular practice. J Neurosurg.

[73] Roa JA, Sabotin RP, Varon A, Raghuram A, Patel D, Morris TW, Ishii D, Lu Y, Hasan DM, Samaniego EA (2021). Performance of aneurysm wall enhancement compared with clinical predictive scales: PHASES, ELAPSS, and UIATS. World Neurosurg.

[74] Robertson AM, Duan X, Aziz KM, Hill MR, Watkins SC, Cebral JR (2015). Diversity in the strength and structure of unruptured cerebral aneurysms. Ann Biomed Eng.

[75] Rousseau O, Karakachoff M, Gaignard A, Bellanger L, Bijlenga P, Constant Dit Beaufils P, L'Allinec V, Levrier O, Aguettaz P, Desilles JP, Michelozzi C, Marnat G, Vion AC, Loirand G, Desal H, Redon R, Gourraud PA, Bourcier R, Investigators I (2021). Location of intracranial aneurysms is the main factor associated with rupture in the ICAN population. J Neurol Neurosurg Psychiatry.

[76] Samaniego EA, Roa JA, Hasan D (2019). Vessel wall imaging in intracranial aneurysms. J Neurointerv Surg.

[77] Sanchez van Kammen M, Greving JP, Kuroda S, Kashiwazaki D, Morita A, Shiokawa Y, Kimura T, Cognard C, Januel AC, Lindgren A, Koivisto T, Jaaskelainen JE, Ronkainen A, Pyysalo L, Ohman J, Rahi M, Kuhmonen J, Rinne J, Leemans EL, Majoie CB, Vandertop WP, Verbaan D, Roos Y, Berg RVD, Boogaarts HD, Moudrous W, Wijngaard I, Hove LT, Teo M, George EJS, Hackenberg KAM, Abdulazim A, Etminan N, Rinkel GJE, Vergouwen MDI (2019). External validation of the ELAPSS score for prediction of unruptured intracranial aneurysm growth risk. J Stroke.

[78] Sang C, Kallmes DF, Kadirvel R, Durka MJ, Ding YH, Dai D, Watkins SC, Robertson AM (2021). Adaptive remodeling in the elastase-induced rabbit aneurysms. Exp Mech.

[79] Santarosa C, Cord B, Koo A, Bhogal P, Malhotra A, Payabvash S, Minja FJ, Matouk CC (2020). Vessel wall magnetic resonance imaging in intracranial aneurysms: principles and emerging clinical applications. Interv Neuroradiol.

[80] Schatlo B, Fung C, Stienen MN, Fathi AR, Fandino J, Smoll NR, Zumofen D, Daniel RT, Burkhardt JK, Bervini D, Marbacher S, Reinert M, Alonzo DD, Ahlborn P, Mendes Pereira V, Roethlisberger M, Seule M, Kerkeni H, Remonda L, Weyerbrock A, Woernle K, Venier A, Perren F, Sailer M, Robert T, Rohde V, Schöni D, Goldberg J, Nevzati E, Diepers M, Gralla J, Z’Graggen W, Starnoni D, Woernle C, Maldaner N, Kulcsar Z, Mostaguir K, Maduri R, Eisenring C, Bernays R, Ferrari A, Dan-Ura H, Finkenstadt S, Gasche Y, Sarrafzadeh A, Jakob SM, Corniola M, Baumann F, Regli L, Levivier M, Hildebrandt G, Landolt H, Mariani L, Guzman R, Beck J, Raabe A, Keller E, Bijlenga P, Schaller K (2021). Incidence and outcome of aneurysmal subarachnoid hemorrhage: the Swiss Study on Subarachnoid Hemorrhage (Swiss SOS). Stroke.

[81] Shikata F, Shimada K, Sato H, Ikedo T, Kuwabara A, Furukawa H, Korai M, Kotoda M, Yokosuka K, Makino H, Ziegler EA, Kudo D, Lawton MT, Hashimoto T (2019). Potential influences of gut microbiota on the formation of intracranial aneurysm. Hypertension.

[82] Shimizu K, Imai H, Kawashima A, Okada A, Ono I, Miyamoto S, Kataoka H, Aoki T (2021) Induction of CCN1 in growing saccular aneurysms: a potential marker predicting unstable lesions. J Neuropathol Exp Neurol. 10.1093/jnen/nlab037 10.1093/jnen/nlab037 33885814

[83] Shimizu K, Kataoka H, Imai H, Yamamoto Y, Yamada T, Miyata H, Koseki H, Abekura Y, Oka M, Kushamae M, Ono I, Miyamoto S, Nakamura M, Aoki T (2021). Hemodynamic force as a potential regulator of inflammation-mediated focal growth of saccular aneurysms in a rat model. J Neuropathol Exp Neurol.

[84] Shimizu K, Miyata H, Abekura Y, Oka M, Kushamae M, Kawamata T, Mizutani T, Kataoka H, Nozaki K, Miyamoto S, Aoki T (2019). High-fat diet intake promotes the enlargement and degenerative changes in the media of intracranial aneurysms in rats. J Neuropathol Exp Neurol.

[85] Shimonaga K, Matsushige T, Ishii D, Sakamoto S, Hosogai M, Kawasumi T, Kaneko M, Ono C, Kurisu K (2018). Clinicopathological insights from vessel wall imaging of unruptured intracranial aneurysms. Stroke.

[86] Smedley A, Yusupov N, Almousa A, Solbach T, Toma AK, Grieve JP (2018). Management of incidental aneurysms: comparison of single Centre multi-disciplinary team decision making with the unruptured incidental aneurysm treatment score. Br J Neurosurg.

[87] Soldozy S, Norat P, Elsarrag M, Chatrath A, Costello JS, Sokolowski JD, Tvrdik P, Kalani MYS, Park MS (2019). The biophysical role of hemodynamics in the pathogenesis of cerebral aneurysm formation and rupture. Neurosurg Focus.

[88] Staarmann B, Smith M, Prestigiacomo CJ (2019). Shear stress and aneurysms: a review. Neurosurg Focus.

[89] Starke RM, Thompson JW, Ali MS, Pascale CL, Martinez Lege A, Ding D, Chalouhi N, Hasan DM, Jabbour P, Owens GK, Toborek M, Hare JM, Dumont AS (2018). Cigarette smoke initiates oxidative stress-induced cellular phenotypic modulation leading to cerebral aneurysm pathogenesis. Arterioscler Thromb Vasc Biol.

[90] Stienen MN, Visser-Meily JM, Schweizer TA, Hanggi D, Macdonald RL, Vergouwen MDI, Unruptured Intracranial A, Investigators SCP (2019). Prioritization and timing of outcomes and endpoints after aneurysmal subarachnoid hemorrhage in clinical trials and observational studies: proposal of a multidisciplinary research group. Neurocrit Care.

[91] Tada Y, Wada K, Shimada K, Makino H, Liang EI, Murakami S, Kudo M, Shikata F, Pena Silva RA, Kitazato KT, Hasan DM, Kanematsu Y, Nagahiro S, Hashimoto T (2014). Estrogen protects against intracranial aneurysm rupture in ovariectomized mice. Hypertension.

[92] Texakalidis P, Hilditch CA, Lehman V, Lanzino G, Pereira VM, Brinjikji W (2018). Vessel wall imaging of intracranial aneurysms: systematic review and meta-analysis. World Neurosurg.

[93] Texakalidis P, Sweid A, Mouchtouris N, Peterson EC, Sioka C, Rangel-Castilla L, Reavey-Cantwell J, Jabbour P (2019). Aneurysm formation, growth, and rupture: the biology and physics of cerebral aneurysms. World Neurosurg.

[94] Tulamo R, Frosen J, Hernesniemi J, Niemela M (2018). Inflammatory changes in the aneurysm wall: a review. J Neurointerv Surg.

[95] Tutino VM, Poppenberg KE, Li L, Shallwani H, Jiang K, Jarvis JN, Sun Y, Snyder KV, Levy EI, Siddiqui AH, Kolega J, Meng H (2018). Biomarkers from circulating neutrophil transcriptomes have potential to detect unruptured intracranial aneurysms. J Transl Med.

[96] Usselman CW, Yarovinsky TO, Steele FE, Leone CA, Taylor HS, Bender JR, Stachenfeld NS (2019). Androgens drive microvascular endothelial dysfunction in women with polycystic ovary syndrome: role of the endothelin B receptor. J Physiol.

[97] Vergouwen MDI, Backes D, van der Schaaf IC, Hendrikse J, Kleinloog R, Algra A, Rinkel GJE (2019). Gadolinium enhancement of the aneurysm wall in unruptured intracranial aneurysms is associated with an increased risk of aneurysm instability: a follow-up study. AJNR Am J Neuroradiol.

[98] Vlak MH, Algra A, Brandenburg R, Rinkel GJ (2011). Prevalence of unruptured intracranial aneurysms, with emphasis on sex, age, comorbidity, country, and time period: a systematic review and meta-analysis. Lancet Neurol.

[99] Wadghiri YZ, Hoang DM, Leporati A, Gounis MJ, Rodriguez-Rodriguez A, Mazzanti ML, Weaver JP, Wakhloo AK, Caravan P, Bogdanov AA (2018). High-resolution imaging of myeloperoxidase activity sensors in human cerebrovascular disease. Sci Rep.

[100] Wang GX, Wen L, Lei S, Ran Q, Yin JB, Gong ZL, Zhang D (2018). Wall enhancement ratio and partial wall enhancement on MRI associated with the rupture of intracranial aneurysms. J Neurointerv Surg.

[101] Wang S, Zhang H, Liu Y, Li L, Guo Y, Jiao F, Fang X, Jefferson JR, Li M, Gao W, Gonzalez-Fernandez E, Maranon RO, Pabbidi MR, Liu R, Alexander BT, Roman RJ, Fan F (2020). Sex differences in the structure and function of rat middle cerebral arteries. Am J Physiol Heart Circ Physiol.

[102] Watton PN, Selimovic A, Raberger NB, Huang P, Holzapfel GA, Ventikos Y (2011). Modelling evolution and the evolving mechanical environment of saccular cerebral aneurysms. Biomech Model Mechanobiol.

[103] Wong GKC, Daly JJ, Rhoney DH, Broderick J, Ogilvy C, Roos YB, Siddiqui A, Torner J, Unruptured Intracranial A, Investigators SCP (2019). Common data elements for unruptured intracranial aneurysm and subarachnoid hemorrhage clinical research: recommendations from the working group on long-term therapies. Neurocrit Care.

[104] Xiao W, Qi T, He S, Li Z, Ou S, Zhang G, Liu X, Huang Z, Liang F (2018). Low wall shear stress is associated with local aneurysm wall enhancement on high-resolution MR vessel wall imaging. AJNR Am J Neuroradiol.

[105] Xu Z, Rui YN, Hagan JP, Kim DH (2019). Intracranial aneurysms: pathology, genetics, and molecular mechanisms. Neuromolecular Med.

[106] Yamashiro Y, Yanagisawa H (2020). The molecular mechanism of mechanotransduction in vascular homeostasis and disease. Clin Sci (Lond).

[107] Zhang M, Peng F, Tong X, Feng X, Li Y, Chen H, Niu H, Zhang B, Song G, Li Y, Liu P, Liu A, Li R (2021) Associations between haemodynamics and wall enhancement of intracranial aneurysm. Stroke Vasc Neurol. 10.1136/svn-2020-000636 10.1136/svn-2020-000636 PMC8485248 33637615

[108] Zhang Y, Fu Q, Wang Y, Cheng J, Ren C, Guan S, Zhu C (2020). Qualitative and quantitative wall enhancement analyses in unruptured aneurysms are associated with an increased risk of aneurysm instability. Front Neurosci.

[109] Zhong W, Su W, Li T, Tan X, Chen C, Wang Q, Wang D, Su W, Wang Y (2021). Aneurysm wall enhancement in unruptured intracranial aneurysms: a histopathological evaluation. J Am Heart Assoc.

